# Plasmonic Nanostructure Biosensors: A Review

**DOI:** 10.3390/s23198156

**Published:** 2023-09-28

**Authors:** Huimin Wang, Tao Wang, Xuyang Yuan, Yuandong Wang, Xinzhao Yue, Lu Wang, Jinyan Zhang, Jian Wang

**Affiliations:** 1 Wuhan National Laboratory for Optoelectronics, Huazhong University of Science and Technology, Wuhan 430074, China; whm@hust.edu.cn (H.W.); xuyang_yuan@hust.edu.cn (X.Y.); wangyuandong@hust.edu.cn (Y.W.); xinzhaoyue@hust.edu.cn (X.Y.); louisewang@hust.edu.cn (L.W.); jy_zhang@hust.edu.cn (J.Z.); 2 Optics Valley Laboratory, Wuhan 430074, China

**Keywords:** plasmonic nanostructure biosensors, surface plasmon resonance, plasmon nanoruler, surface-enhanced Raman scattering

## Abstract

Plasmonic nanostructure biosensors based on metal are a powerful tool in the biosensing field. Surface plasmon resonance (SPR) can be classified into localized surface plasmon resonance (LSPR) and propagating surface plasmon polariton (PSPP), based on the transmission mode. Initially, the physical principles of LSPR and PSPP are elaborated. In what follows, the recent development of the biosensors related to SPR principle is summarized. For clarity, they are categorized into three groups according to the sensing principle: (i) inherent resonance-based biosensors, which are sensitive to the refractive index changes of the surroundings; (ii) plasmon nanoruler biosensors in which the distances of the nanostructure can be changed by biomolecules at the nanoscale; and (iii) surface-enhanced Raman scattering biosensors in which the nanostructure serves as an amplifier for Raman scattering signals. Moreover, the advanced application of single-molecule detection is discussed in terms of metal nanoparticle and nanopore structures. The review concludes by providing perspectives on the future development of plasmonic nanostructure biosensors.

## 1. Introduction

**Figure 1:**
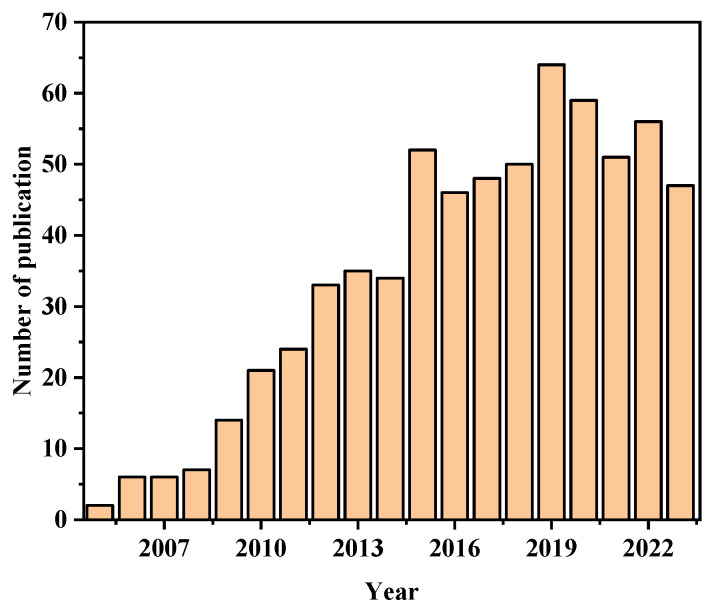
Number of publication as a function of year for the plasmonic nanostructure biosensors. The data are from Scopus.

In the past few decades, noble metals have shown unique optical properties at the visible and near infrared region. The applications of nanotechnology are becoming more and more extensive, such as ultrasensitive molecule detection [1], quantum optics [2], photovoltaics [3], and optoelectronics [4]. Plasmonics, an emerging field, is currently undergoing rapid development. In fact, plasmonic sensing was recognized as one of the top ten emerging technologies by Scientific American in 2018 [5]. Plasmonic nanostructure sensors, based on metal, find extensive utilization in various fields, including biological analysis, disease diagnosis, environmental monitoring, and food safety. In the biosensing field, these sensors are known for their highly sensitive, rapid, real-time, and label-free operation [6,7,8,9,10,11] in comparison with fluorescence analysis [12,13] and electrochemical analysis [14,15]. In the last twenty years, there has been a steady increase in the number of publications focusing on plasmonic nanostructure biosensors as shown in Figure 1. The objective of this study is to improve the understanding of the emerging field of research and to clarify the important characteristics of nanostructured surfaces in relation to their biosensing application.

Surface plasmon resonance (SPR) can be excited by the incident light at the interface between a metal and a dielectric. The electromagnetic field is confined to the subwavelength scales and has electric field enhancement properties. It is possible to manipulate light at the nanoscale, which is far below the diffraction limit. SPR can be classified into localized surface plasmon resonance (LSPR) and propagating surface plasmon polariton (PSPP) according to the transmission mode. LSPR usually occurs in metal nanoparticles [16,17], such as nanospheres, nanoribbons, nanodisks, nanorings, nanocrosses and so on. LSPR can result in strong absorption, scattering, reflection, or transmission at specific wavelengths, which are highly influenced by the morphology and dimensions of the nanoparticle, as well as the local surroundings [18]. PSPP is usually excited on a flat metal film, which is easier to manufacture and use. LSPR-based and PSPP-based biosensors are inherently sensitive to the refractive index changes of the surroundings, which means that they are ideal candidates for plasmonic biosensors.

The coupling of LSPR between two metal nanoparticles in close proximity leads to the generation of a light-scattering spectrum that is highly dependent on the distance between the nanoparticles. This effect has been utilized in the creation of plasmon nanorulers [19], which possess the capability to measure distances at the nanoscale in one dimension. Plasmon nanorulers that rely on sensitive colorimetric schemes have been used to monitor DNA hybridization [20]. Plasmon nanorulers, unlike molecular rulers based on dye-pair fluorescence resonance energy transfer [21], offer exceptional photo stability and brightness due to the incorporation of noble metal nanoparticles. Furthermore, it is possible to position multiple nanoparticles in close proximity to each other [22,23,24]. Colorimetric biosensors that rely on metal nanoparticles are suitable options when prioritizing quickness and ease of use over extremely high sensitivity and accuracy. These biosensors have the ability to generate a visual response.

For the accuracy and specificity of the results, complex biological modification steps are required in the inherent SPR technique and plasmon nanoruler technique. The surface-enhanced Raman scattering (SERS) technique can directly provide the fingerprint information of molecules to be measured, which can determine their compositions and concentrations. There is now a consensus among researchers that enhanced Raman scattering results from a combination of electromagnetic enhancement associated with LSPR in the metal nanoparticles acting as the SERS substrate, along with a chemical enhancement [25,26,27]. In general, the electromagnetic enhancement factor contributes more to Raman scattering enhancement (up to 10^10^), which requires molecules within a few nanometers (1–10 nm) from the surface of the SERS substrate. In contrast, chemical enhancement necessitates the sub-nanometer separation distances, such as contact or a few Angstroms, to generate a chemical enhancement factor of 10^2^–10^4^ [28]. The electric field enhancement of LSPR is significantly important for SERS spectroscopy.

**Figure 2:**
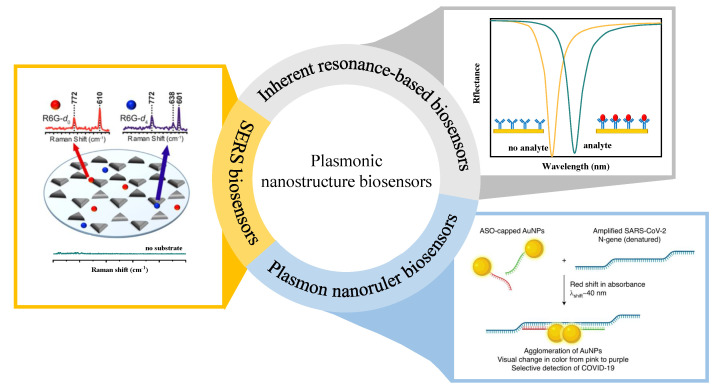
Classification of plasmonic nanostructure biosensors, including inherent resonance-based biosensors, plasmon nanoruler biosensors, and SERS biosensors. Insets are representative examples. Left panel: Raman scattering enhancement of the analyte (R6G) in the presence of nanostructure (SERS substrate). Reproduced from Ref. [89] with permission. Copyright © 2013 American Chemical Society. Upper right panel: PSPP peak shift at the presence of the analyte. Lower right panel: Plasmon nanoruler composed of ASO-cappped AuNPs and amplified SARS-CoV-2 N-gene that can change the distance between AuNPs. Reproduced from Ref. [78] with permission. Copyright © 2021, The Author(s), under exclusive license to Springer Nature Limited.

Our review is structured into six main parts. The first part (Section 2) focuses on illustrating the physical principles of LSPR and PSPP. The second part of the review (Section 3) discusses the parameters that can characterize the performance of a biosensor. The last three parts (Section 4, Section 5 and Section 6) review three categories of plasmonic nanostructure biosensors with different sensing principles in Figure 2. The corresponding illustrations show the representative examples. The upper right panel shows the inherent resonance-based biosensors [9,29,30,31,32,33,34,35,36,37,38,39,40,41,42,43,44,45,46,47,48,49,50,51,52,53,54,55,56,57,58,59,60,61,62,63,64]. The corresponding illustration is the PSPP-based refractive index biosensors. When the analyte is captured, the resonant peak is redshifted, and then the analyte is detected. The lower right panel shows the plamon nanoruler biosensors [19,65,66,67,68,69,70,71,72,73,74,75,76,77,78,79,80,81,82]. The plasmon nanoruler is composed of the ASO-capped gold nanoparticles (AuNPs) and amplified SARS-CoV-2 N-gene that can change the distance between AuNPs [78]. The left panel shows the SERS biosensors [83,84,85,86,87,88,89,90,91,92,93,94,95,96]. The corresponding illustration [89] shows that the nanostructure acts as a Raman scattering signal amplification. When the nanostructure (SERS substrate) is absent or present, the Raman scattering signal of rhodamine 6G (R6G) is weak or significant, respectively. The last part (Section 7) discusses the application of the single-molecule detection in terms of metal nanoparticle and nanopore structures. In the subsequent discussion, we establish a direct correlation between the fundamental optical properties of SPR and their notable biosensing applications.

## 2. Basic Principles of Surface Plasmon 

The term plasmon describes the collective oscillation of electrons within a metal, which can be understood as a quasiparticle from a quantum perspective. The surface plasmon is a special type that occurs at the interface between a metal and a dielectric. SPR is an optical phenomenon that occurs when light is incident on a metal surface at (or near) the plasma frequency. In other words, SPR is generated by the excitation of surface plasmon through incident light. In what follows, the physical mechanisms of LSPR and PSPP are discussed in Section 2.1 and Section 2.2, respectively.

### 2.1. Physics of LSPR

**Figure 3:**
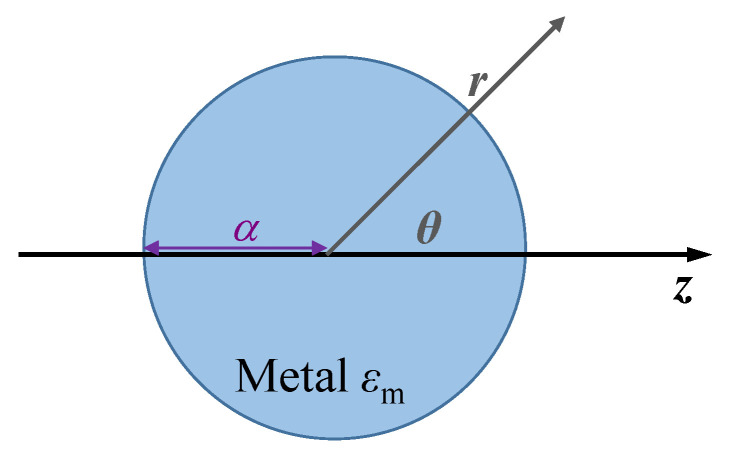
Structure diagram of a metal spherical nanoparticle. *a* represents the radius, θ is the angle to the *z* axis, *r* is the distance from the center of the nanosphere, and $\varepsilon_{m}$ is the permittivity of the metal nanosphere.

LSPR does not propagate, which generally exists around the subwavelength distance near conductive nanoparticles. When an incident light illuminates metal nanoparticles, such as gold and silver, the electric field of the incident light can cause the electrons of the conduction band to collectively oscillate. There is an obvious binding force at the surface of the nanosphere, which causes resonance and amplifies the electric field in the near field region of the inner and outer layers of the nanosphere. The property can be used for SERS to detect molecules [16]. As illustrated in Figure 3, we consider the simplest metal spherical nanoparticle model to analyze the physical mechanism of LSPR.

When the radius *a* of the nanosphere is much smaller than the incident light wavelength λ (i.e., a/λ<0.1), the electric field is static around the nanosphere. Therefore, a quasi-static approximation can be used to solve Maxwell’s equations. The electric potentials inside $\Phi_{\mathrm{in}}$ and outside $\Phi_{\mathrm{out}}$ the nanosphere satisfy the equations as follows [97]:

(1) $$\nabla^{2}\Phi_{\mathrm{in}}=0\;,r<a$$

(2) $$\nabla^{2}\Phi_{out}=0,\;r>a$$

where *r* is the distance from the center of the nanosphere. The electric potential is continuous at the interface between the nanosphere and surroundings. The electric potentials inside $\Phi_{\mathrm{in}}$ and outside $\Phi_{\mathrm{out}}$ the nanosphere can be expressed as

(3) $$\Phi_{\mathrm{in}}\left(r,\theta \right)=-\frac{3\varepsilon_{d}}{\varepsilon_{m}+2\varepsilon_{d}}E_{0}r\cos\theta ,r<a$$

(4) $$\Phi_{\mathrm{out}}\left(r,\theta \right)=-E_{0}r\cos\theta +\frac{\varepsilon_{m}-2\varepsilon_{d}}{\varepsilon_{m}+2\varepsilon_{d}}E_{0}r_{0}^{3}\frac{\cos\theta }{r^{2}}=-E_{0}r\cos\theta +\frac{pr}{4\pi \varepsilon_{0}\varepsilon_{d}r^{3}},r>a$$

where $E_{0}$ is the electric field of the incident light, θ is the angle to the *z* axis, *p* is the electric dipole momentum, and $\varepsilon_{m}=\varepsilon_{m}^{\prime }+i\varepsilon_{m}^{\prime \prime }$ and $\varepsilon_{d}$ are the permittivities of the nanosphere and the surroundings, respectively. We can obtain the polarizability α of the nanosphere as follows:

(5) $$\alpha =\frac{p}{\varepsilon_{0}\varepsilon_{d}E_{0}}=4\pi r_{0}^{3}\frac{\varepsilon_{m}-2\varepsilon_{d}}{\varepsilon_{m}+2\varepsilon_{d}}$$

When $\varepsilon_{m}$ and $\varepsilon_{d}$ satisfy the relationship of $\varepsilon_{m}+2\varepsilon_{d}=0$, the polarizability α is the maximum and the nanosphere has the strongest resonance response to the incident light. Equation (5) shows that LSPR is inherently sensitive to the refractive index changes of the surroundings. We can calculate scattering cross-section $C_{\mathrm{sca}}$, the extinction cross-section $C_{\mathrm{ext}}$, and absorption cross-section $C_{\mathrm{abs}}$ as follows:

(6) $$C_{\mathrm{sca}}=\frac{k^{4}}{6\pi }{\left|\alpha \right|}^{2}=\frac{8\pi }{3}k^{4}a^{6}{\left|\frac{\varepsilon_{m}-2\varepsilon_{d}}{\varepsilon_{m}+2\varepsilon_{d}}\right|}^{2}$$

(7) Cext=kIm(α)=4πka3Imεm−2εdεm+2εd

(8) $$C_{\mathrm{abs}}=C_{\mathrm{ext}}-C_{\mathrm{sca}}$$

where *k* is the wave vector of the incident light. The extinction cross-section $C_{\mathrm{ext}}$ and scattering cross-section $C_{\mathrm{sca}}$ are proportional to $a^{3}$ and $a^{6}$, respectively. It shows that the resonance absorption predominates for the smaller nanospheres, and the resonance scattering predominates for the lager nanospheres. When *a* is 80 nm, this transition occurs [98]. The electrons collectively oscillate when LSPR occurs, and the scattering and absorption of the nanospheres can be enhanced. The characteristics can be applied to refractive index sensing and plasmon nanoruler sensing [16].

### 2.2. Physics of PSPP

**Figure 4:**
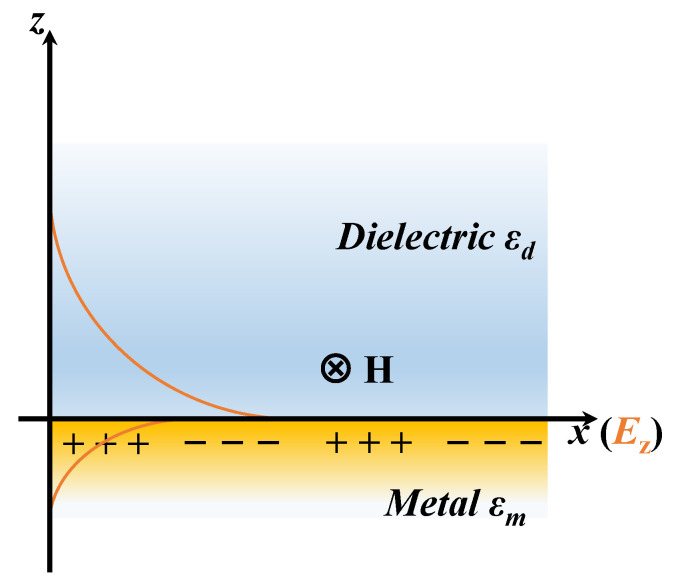
Surface charges at the interface when PSPP is excited by the transverse magnetic wave. The orange solid lines show that electromagnetic waves decay exponentially into both the metal and dielectric parts.

PSPP is an oscillation of charge density that occurs at the interface between a metal and a dielectric with opposite signs of dielectric constants [99]. We consider the simplest geometry consisting of a semi-infinite metal and a semi-infinite dielectric in Figure 4. The dielectric constant of the metal is expressed as $\varepsilon_{m}=\varepsilon_{m}^{\prime }+i\varepsilon_{m}^{\prime \prime }$. The dielectric constant of the dielectric is denoted as $\varepsilon_{d}$. As there is no boundary perpendicular to $E_{x}$, this component remains conserved across the boundary. The component $D_{z}$ is continuous, and the component $E_{z}$ changes, resulting from different dielectric constants on both sides of the interface. It is evident that a transverse electric wave (i.e., s-polarized wave) does not lead to the formation of charges at the planar interface. However, a transverse magnatic wave (i.e., p-polarized wave) will inevitably generate time-dependent polarization charges at the interface.

We only consider the transverse magnetic wave incidence below, and the *x*-*y* plane is treated as the interface. The electromagnetic field distribution is expressed [100,101]; when *z* > 0, one has

(9) $$E_{1}\left(x,z,t\right)=-\frac{A}{i\omega \varepsilon_{d}\varepsilon_{0}}\left(k_{1z},0,ik\right)\exp\left(ikx-k_{1z}z-i\omega t\right)$$

(10) $$H_{1}\left(x,z,t\right)=\left(0,A,0\right)\exp\left(ikx-k_{1z}z-i\omega t\right)$$

when *z* < 0, one has

(11) $$E_{2}\left(x.z,t\right)=-\frac{B}{i\omega \varepsilon_{m}\varepsilon_{0}}\left(-k_{2z},0,ik\right)\exp\left(ikx+k_{2z}z-i\omega t\right)$$

(12) $$H_{2}\left(x.z,t\right)=\left(0,B,0\right)\exp\left(ikx+k_{2z}z-i\omega t\right)$$

where

(13) $$k_{1z}=\sqrt{k^{2}-\varepsilon_{d}{\left(\omega /c\right)}^{2}}$$

(14) $$k_{2z}=\sqrt{k^{2}-\varepsilon_{m}{\left(\omega /c\right)}^{2}}$$

which illustrates that the electromagnetic fields decay exponentially as the distance from the interface increases.

When *z* = 0, there are

(15) A=B

(16) $$A\frac{k_{1z}}{\varepsilon_{d}}=-B\frac{k_{2z}}{\varepsilon_{m}}$$

We can obtain the dispersion relation of PSPP, as follows:

(17) $$\frac{k_{2z}}{k_{1z}}=-\frac{\varepsilon_{m}}{\varepsilon_{d}}$$

(18) $$k_{pspp}=\frac{\omega }{c}\sqrt{\frac{\varepsilon_{d}\varepsilon_{m}}{\varepsilon_{d}+\varepsilon_{m}}}={k^{\prime }}_{pspp}+i{k^{\prime \prime }}_{pspp}$$

Equation (18) shows that PSPP is inherently sensitive to the refractive index changes of the dielectric. The wave vector of PSPP $k_{pspp}$ is greater than that of vacuum *k* at the same frequency. PSPP can be excited by the incident light with additional momentum or energy that has the same polarization state as PSPP. The real part of the wave vector $k_{pspp}^{\prime }$ is connected to the effective refractive index of PSPP, and the imaginary part of the wave vector $k_{pspp}^{\prime \prime }$ is linked to the attenuation of PSPP in the direction of propagation. The propagating length $L_{pspp}$ along *x*-axis is determined by $k_{pspp}^{\prime \prime }$ and determines the maximum length of the nanophotonics device based on PSPP. It can be expressed as

(19) $$L_{pspp}=\frac{1}{2{k^{\prime \prime }}_{pspp}}$$

where $L_{pspp}$ greatly exceeds the wavelength of the incident light.

Figure 4 shows charge density oscillations that propagate on the *x*-axis. The electromagnetic waves decay exponentially into both the metal and dielectric in Figure 4 (orange solid line), and the intensity reaches a maximum at the *x*-*y* plane [102]. The penetration depth of the evanescent field in the dielectric $\delta_{d}$ and metal $\delta_{m}$ can be expressed as [103]

(20) $$\delta_{d}=\frac{1}{k_{0}}\left|\frac{\varepsilon_{d}+{\varepsilon^{\prime }}_{m}}{\varepsilon_{d}^{2}}\right|$$

(21) $$\delta_{m}=\frac{1}{k_{0}}\left|\frac{\varepsilon_{d}+{\varepsilon^{\prime }}_{m}}{\varepsilon_{r}^{2}}\right|$$

where $\delta_{d}$ is on the order of the light wavelength, which determines the thickness range that is sensitive to the changes of the dielectric refractive index.

Next, we make a comprehensive comparison between LSPR and PSPP from the aspects of the excitation methods, wavelength tunability, visual sensing, sensitivity, and electric field enhancement. When the light directly interacts with the metallic nanoparticles, LSPR is excited. PSPP is excited at the interface between the smooth metal film and the dielectric with the help of the prism or other components. In comparison with PSPP biosensors, LSPR biosensors offer advantages in terms of the ease of miniaturization, flexibility in design, and lower fabrication costs. The PSPP wavelength can be conveniently adjusted across the visible and near-infrared regions by changing the incident angle. However, the adjustment of the LSPR wavelength needs to change the shape or size of the metal nanoparticles. Gold nanoparticle solutions with an exceptionally high extinction coefficient can facilitate colorimetric sensing that is visible to the naked eye. In contrast, the gold film lacks this capability. Compared with PSPP, LSPR has broader resonance peaks due to the strong radiative damping [104,105,106]. The figure of merit (FOM) of the PSPP biosensors is significantly higher, ranging from one to two orders of magnitude higher than that of LSPR. The bulk sensitivity of the PSPP biosensors can reach an order of magnitude of $10^{4}$ nm/RIU and is two orders of magnitude higher than that of LSPR due to their longer penetration depth [9]. LSPR has a greater local electric field enhancement in the 5–15 nm range [107] and theoretically obtains a higher surface sensitivity [108].

## 3. Performance Characteristics of Plasmonic Bioensors

Biosensors based on plasmonic nanostructures are extensively investigated in current research studies for detecting changes in the environment, such as molecule-binding events. Surface plasmons are able to confine light to the dimensions that are much smaller than the diffraction limit, which is achieved through the coupling between the incident light and the collective oscillation of the surface free electrons. This unique feature of SPR allows it to overcome the diffraction limit of conventional optics and enables the fabrication of biosensors at a subwavelength scale [109,110]. Plasmonic nanostructure biosensors are a powerful platform for optical sensing applications. The optical sensors can provide various physical parameters that can be measured, such as optical intensity, resonance wavelength, incident angle, phase, and polarization. Such a feature implies that the optical sensors can be suitable for different situations to monitor the molecule changes.

The following characteristic parameters are used to assess the sensing performance, which include full width at half maximum (FWHM), FOM, sensitivity (*S*), and limit of detection (LOD). FWHM and FOM are used to evaluate the resolution of the spectrum. The lower the FWHM and the higher the FOM, the higher the signal-to-noise ratio in the experiment. Sensitivity is the most intuitive parameter to characterize the biosensor performance and is defined as the ratio of the output changes (e.g., optical intensity, resonance wavelength, and incident angle) to target changes to be detected (e.g., refractive index). Specifically, the inherent resonance-based plasmonic biosensors are sensitive to the refractive index changes in the surroundings. When biosensors monitor the resonant wavelength at a fixed incident angle, *S* and FOM can be expressed as

(22) S=Δλ/Δn, FOM=S/FWHM

where Δλ is the resonant wavelength shifts of reflected or transmitted spectra corresponding to the changes in the refractive index Δn. For biosensors based on the intensity detection, *S* and FOM are defined as [111]

(23) $$S=\Delta I/\Delta n,\;FOM=S/I_{ref}$$

where ΔI is the intensity changes of the spectra with the refractive index changes Δn, and $I_{ref}$ is the resonance intensity of the reference point. LOD refers to the smallest amount of molecules that a biosensor can detect and can be expressed as

(24) LOD=3σ/S

where σ is the standard deviation of the biosensor output for a blank sample. When different kinds of biosensors detect the same molecules, LOD is a common parameter to evaluate the performance of the biosensors.

## 4. Inherent Resonance-Based Biosensing

The refractive index changes of the dielectric surroundings result in shifts of the resonance peak. Generally, the penetration depths of LSPR and PSPP are around 5–15 nm [112] and around 200–300 nm [113], respectively. When the refractive index varies in different regions within the penetration depth, the inherent resonance-based biosensors can be utilized to measure the sensitivities of the bulk refractive index and the molecule binding events. We classify the inherent resonance-based biosensors into three subcategories: (i) localized plasmonic eigenmodes biosensors in Section 4.1, (ii) propagating plasmonic eigenmodes biosensors in Section 4.2, and (iii) coupled propagating–localized plasmonic biosensors in Section 4.3.

### 4.1. Localized Plasmonic Eigenmode Biosensors

LSPR happens when the electric field of the incident light matches with the electrons oscillating at the surface of the metal nanoparticles [29,114]. Gold possesses outstanding intrinsic properties that include biocompatibility [115], exceptional chemical stability [116], and easy surface functionalization by the S-Au bound [117]. Gold nanoparticles have been widely studied in LSPR biosensors due to the advantages mentioned above.

#### 4.1.1. Metal Nanoparticle-Based LSPR Biosensors

The characteristic optical properties of the nanoparticles originate from LSPR [114,118] and depend on their size, aspect ratio, shape, and the refractive index of the surroundings [119,120]. For more complex shape nanoparticles, the extinction cross-section $C_{\mathrm{ext}}$ can be modified as

(25) $$C_{\mathrm{ext}}=\frac{24\pi^{2}a^{3}\sqrt{\varepsilon_{d}^{3}}N}{\lambda \ln\left(10\right)}\frac{\varepsilon^{\prime \prime }}{\left(\varepsilon^{\prime }+\chi \varepsilon_{d}\right)+{\varepsilon^{\prime \prime }}^{2}}$$

where λ is the incident light wavelength, *N* is the electron density, and χ is a variable that depends on the aspect ratio of the nanoparticles. As shown in Equation (25), these factors influence the absorption and scattering processes. The significant impact of the refractive index changes of the surroundings on the spectra has been extensively studied in the field of LSPR sensing. With the development of advanced nanoparticle synthesis techniques, nanobranch, nanorod, and nanobipyradmid, and so on have been synthesized. The role of the nanoparticle shape in enhancing LSPR signals has been observed.

Nanoparticles with sharp tips can generate a stronger electric field and achieve higher sensitivity, which is attributed to electrons gathering near the tips [29]. In addition, Xu et al. reviewed that nanoparticles with high symmetry do not exhibit high sensitivity. When the shape was altered to be less symmetrical, there was a significant improvement in the electric field intensity [29]. Unfortunately, nanoparticles with less symmetry pose challenges in terms of synthesis and reproducibility.

**Figure 5:**
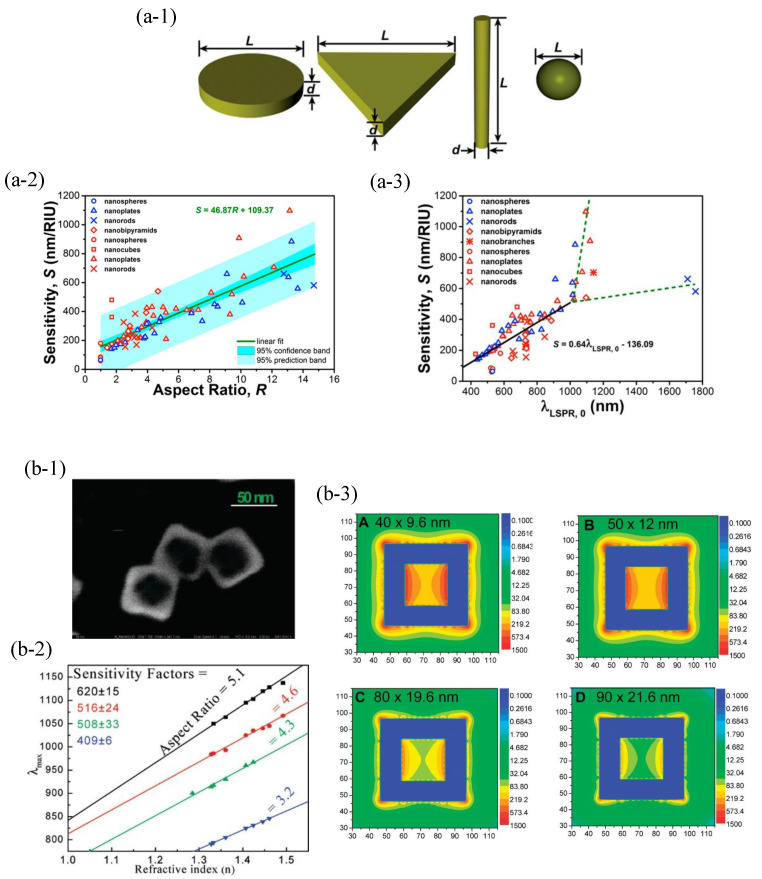
(**a-1**) Aspect ratio (*R* = *L*/*d*) diagram of nanoparticles with different shapes. (**a-2**) Refractive index sensitivity of different nanoparticles with varying aspect ratios *R*. (**a-3**) Sensitivity of different nanoparticles as a function of surface plasmonic resonance wavelength $\lambda_{\mathrm{LSPR},0}$. Reproduced from Ref. [30] with permission. Copyright © 2016 American Chemical Society. (**b-1**) Scanning electron microscope (SEM) image of the gold nanoframes. (**b-2**) Sensitivities of the gold nanoframes at different aspect ratios. (**b-3**) Electric field enhancement contour maps of the gold nanoframes with the same aspect ratio but varying wall lengths and wall thicknesses. Reproduced from Ref. [32] with permission. Copyright © 2010 American Chemical Society.

According to a comprehensive study [30], the correlation between shape and sensitivity was significantly weaker compared to the correlation between the aspect ratio and sensitivity by analyzing over 74 kinds of nanoparticles. As illustrated in Figure 5(a-1), the aspect ratio *R* is defined as *R* = *L*/*d*, in which *L* and *d* are different in various structures. For a nanosphere, *L* = *d* and *R* is always 1. By analyzing all the sensitivities of nanoparticles with various shapes, sizes, compositions, and cross-sectional areas, the relationship between sensitivity *S* and aspect ratio *R* can be fitted as *S* = 46.87*R* + 109.37. There is a linear relationship between *S* and *R* in Figure 5(a-2). The relationship between sensitivity *S* and the initial plasmonic resonance wavelength $\lambda_{\mathrm{LSPR},0}$ is also investigated in Figure 5(a-3). The analysis reveals the trend of sensitivity changes. In such cases, the sensitivity is increased linearly at $\lambda_{\mathrm{LSPR},0}$ below 1000 nm and becomes close to 600 nm/RIU eventually. The linear relationship between *S* and $\lambda_{\mathrm{LSPR},0}$ can be fitted as *S* = 0.64$\lambda_{\mathrm{LSPR},0}-136.09$. This divergence implies that the sensitivity of the nanoparticles is more influenced by the structure parameters rather than the $\lambda_{\mathrm{LSPR},0}$.

In addition, the hollow nanoparticles were also prepared for refractive index sensing and achieved higher sensitivity in comparison with the solid nanoparticles [31,32,33,34,35,36]. As shown in Figure 5(b-1,b-2), the gold nanoframe achieved a sensitivity of around 620 nm/RIU [32]. The sensitivity of nanoparticles depends on the surface plasmonic field strength. Figure 5(b-3) shows that the higher sensitivity of the gold nanoframe results from the resonant coupling between the external and internal plasmonic fields.

However, nanoparticles synthesized by the chemical method are usually disordered on the substrate, which results in spectra widening, FWHM broadening and a reduction in sensing sensitivity. It is important to note that the majority of nanoparticle-based sensors have refractive index sensitivities below 1000 nm/RIU and FOM below 10/RIU [121].

#### 4.1.2. Fano Resonance-Based Biosensors

Fano resonance has an asymmetric spectral line shape, which is attributed to the interference between a broad spectral continuum state and a narrow spectral discrete state [41]. When Fano resonance occurs, there is a distinct narrowed resonance peak in the spectra, which is particularly important to enhance the FOM and improve the resolution of the spectra. Meanwhile, a significant near-field enhancement is generated near the nanostructure, which is beneficial for ultra-sensitive detection. Fano resonance biosensors have been achieved in different nanostructures that supported narrow subradiance and broad radiant modes [37,38,39,40,41,42,43,44,45,122].

For example, a nanostructure array, including a narrow slit and nanohole pairs on the same side of the slit, was fabricated and investigated [41]. Fano resonance originated from the interference effect between the SPP modes of the hole array and the coupled SPP of the narrow slits. The researchers obtained a sensitivity and FOM of approximately 1200 nm/RIU and about 92/RIU in the experiments, respectively. Lee et al. fabricated a capped gold nanoslit array by the thermal-embossing template-stripping method, and investigated the tailorable Fano resonance [43]. The Bloch wave SPPs occurred on the outside surface plasmon wave between nanoslits. The cavity mode resulted from the gap plasmon in the nanoslits. The former and the latter can be treated as a discrete state and a continuum state, respectively, and both of them were coupled to produce a sharp and asymmetric Fano resonance. The biosensor performance can be improved by adjusting the length and width of the nanoslit. FWHM of the Fano mode can be as low as 3.68 nm, and FOM is up to 252/RIU at the optimal structure parameters. Moreover, the sensitivity can be up to 926 nm/RIU with the 1000 nm period and 60 nm width nanoslit. Hsieh et al. [42] combined the capped gold nanoslit array and microfluidic polymerase chain reaction for the first time and successfully detected the DNA sequence of latent membrane protein 1 (LMP1) as low as $∼\;\;10^{-11}$ g/mL. In comparison with the traditional machine, the detection rate was improved by three times. The low-cost manufacturing method of hot-embossing nanoimprinting lithography meant that the device offered the possibility of large-scale production. Ahmed et al. fabricated the optical disc-based metasurfaces that have a large-scale active area with uniform surface patterns and can generate the asymmetric plasmonic modes [40]. This allowed for tunable optical Fano resonance, which can be used in conjunction with a microfluidic channel for multiple target detection. The quantitative detection of protein G, and even whole viral particles of SARS-CoV-2, were achieved. The method showed high sensitivity and specificity. The plasmonic metasurface platform is both cost effective and compact, making it possible for the efficient and real-time detection of SARS-CoV-2 and other pathogens. An overview of the recently reported Fano resonance-based biosensors is reported in Appendix A Table A1.

### 4.2. Propagating Plasmonic Eigenwaves Biosensors

PSPP, being a nonradiative surface wave, is highly responsive to the refractive index changes. The prism-coupled and grating-coupled mechanisms can provide extra momentum to excite PSPP, which is illustrated in Section 4.2.1 and Section 4.2.2, respectively.

#### 4.2.1. Prism-Coupled Mechanism

Under the attenuated internal reflection, the vector-matching condition $n_{p}k_{0}\sin\theta =k_{\mathrm{PSPP}}$ is fulfilled, where θ is the angle of the incident light, $n_{p}$ is the refractive index of the prism, and $k_{0}$ is the wave vector in vacuum. The excited evanescent field overlaps the detected region. The refractive index changes of the detected region can affect the vector-matching condition, leading to changes in the spectra. When the wavelength (or angle) of the incident light is fixed, a dip appears in the reflected spectrum in the angle (or wavelength) scanning mode.

PSPP biosensors are widely studied due to their simple structure, robust optical properties and easy molecule modification. When the refractive index of the entire surroundings changes, the sensitivity of PSPP biosensors can readily achieve higher than 1000 nm/RIU. However, PSPP biosensors with a Kretschmann configuration require bulky instruments and incur high detection costs. This renders PSPP-based biosensors unsuitable for miniaturization and point-of-care (POC) diagnosis. Depositing a dielectric layer onto the gold film results in the observation of plasmon waveguide resonance (PWR). This phenomenon can be utilized for enhancing the performance of sensing [46,47].

In addition, two-dimensional (2D) nanomaterials have been proposed to improve the performance of PSPP biosensors over the last few decades [48,63,123,124,125,126,127,128,129,130,131,132,133]. Two-dimensional nanomaterials have sheet-like structures, which are larger than 100 nm in transverse dimensions and are less than 5 nm in thickness [134]. They have some distinguished characteristics, such as high surface-to-volume ratio and good charge transfer properties, which is conducive to sensing performance improvement.

**Figure 6:**
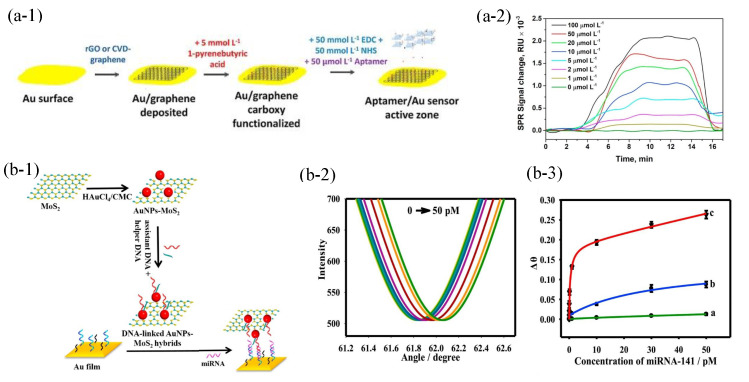
(**a-1**) Fabrication and surface modification schematic of a PSPP biosensor with monolayer graphene. (**a-2**) Signal changes as a function of time at different concentrations (1–100 μM) of kanamycin residues. Reproduced from Ref. [48] with permission. Copyright © 2021 Elsevier B.V. (**b-1**) Schematic of a PSPP biosensor with gold nanoparticles decorated ${\mathrm{MoS}}_{2}$. (**b-2**) Reflection spectra of a PSPP biosensor at different concentrations (0–50 pM) of miRNA-141. (**b-3**) Δθ as a function of the concentration of miRNA-141 for different sensing strategies in which a represented the PSPP biosensor, b represented the PSPP biosensor with gold nanoparticles, and c represented PSPP biosensor with gold nanoparticle-decorated ${\mathrm{MoS}}_{2}$. Reproduced from Ref. [50] with permission. Copyright © 2017 Elsevier B.V.

A monolayer of graphene was deposited on the gold film by the chemical vapor deposition technology in Figure 6(a-1). The biosensor detected the kanamycin residues of the linear concentration range of 1–100 μM in Figure 6(a-2), and the LOD is 285 nM [48]. Graphene-based PSPP biosensors still need to be further explored in terms of mass production, especially with regard to fabrication efficiency [49]. In addition, transition metal dichalcogenide, such as ${\mathrm{MoS}}_{2}$ is also a kind of 2D material. Gold nanoparticle-decorated ${\mathrm{MoS}}_{2}$ was used to improve the PSPP biosensors performance in Figure 6(b-1) [50]. As shown in Figure 6(b-2), the proposed biosensors achieved the detection of 0.5 fM miRNA-141. In comparison with gold nanoparticles (b, blue line), gold nanoparticle-decorated ${\mathrm{MoS}}_{2}$ (c, red line) had a higher biosensing response as shown in Figure 6(b-3). It is attributed to the larger surface area of ${\mathrm{MoS}}_{2}$, which can be used to attach more gold nanoparticles. For PSPP biosensors based on ${\mathrm{MoS}}_{2}$, placing a monolayer on a larger surface area still needs to be explored [49].

When the signs of the dielectric constant components of a material in the direction of the optical axis and the direction perpendicular to optical axis are opposite, a hyperbolic material (HMM) can be constructed. Two classic structures include metal-dielectric multilayer and nanorods array. HMMs have been utilized in the development of biosensors, exhibiting record-breaking performance [9,51,52,53,54,55,56,57]. The optical response of the HMMs depends not only on the plasmonic response of each meta-atom but also on the electromagnetic coupling between meta-atoms when the incident light is p-polarized. HMMs support bulk plasmon polariton (BPP), which has a penetration depth of 500 nm [52]. The excited field is not only localized within a HMM but also has an evanescent field extending into the surroundings, which provides an overlap between the sensing field and the detected molecules. Recently, our research group successfully fabricated a nanorod HMM (NHMM) biosensor by combining electron beam lithography (EBL) and electroplating. The biosensor obtained the record-high sensitivity of 41,600 nm/RIU and FOM of 416/RIU [9]. The improved sensing performance resulted from the more regular arrangement of the fabricated nanorods and the narrower FWHM [135]. It is important to mention that EBL technology is both time consuming and expensive. The utilization of nanoimprint technology has the potential to facilitate large-scale preparation and mass production. An overview of propagating plasmonic eigenwaves biosensors based on the prism-coupled mechanism is reported in Table A2.

#### 4.2.2. Grating-Coupled Mechanism

Although the sensors based on the prism-coupled mechanism can achieve high sensitivity, it is not conducive to integration and miniaturization. To eliminate the prism setup, miniaturized grating-coupled biosensors are illustrated in this section. PSPP biosensors based on the grating-coupled mechanism utilize the diffraction of the incident light to provide the extra wave vectors. The coupling condition is expressed as $k_{0}n_{d}\sin\theta +m\frac{2\pi }{P}=\pm k_{pspp}$, where $k_{0}$ is the wave vector in a vacuum, $n_{d}$ is the refractive index in the incident light region, *m* is an integer, and *P* is the period of the grating. The sensitivity of PSPP biosensors based on the prism-coupled mechanism is higher than that of the grating-coupled mechanism [136]. The sensitivity of PSPP biosensors based on the grating-coupled mechanism is generally lower than 1000 nm/RIU.

**Figure 7:**
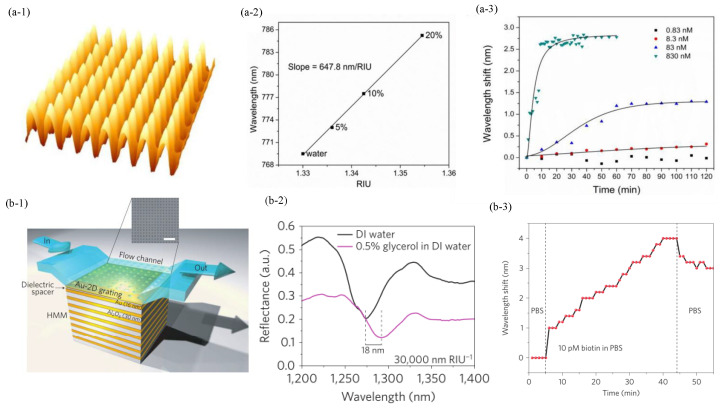
(**a-1**) Diagram of crossed surface relief gratings. (**a-2**) Fitting curve of wavelength as a function of refractive index. The slope represents sensitivity. (**a-3**) Changes of wavelength with time, when different concentrations of streptavidin were measured. Reproduced from Ref. [59] with permission. Copyright © 2017, American Chemical Society. (**b-1**) Schematic of MHMM excited by Au 2D nanohole grating. (**b-2**) Reflection spectra of MHMM for deionized water and 0.5% glycerol in deionized water. (**b-3**) Wavelength shift as a function of time for 10 pM biotin. Reproduced from Ref. [53] with permission. Copyright © 2016, Springer Nature Limited.

The one-dimensional (1D) metal grating is widely recognized as the most common structure to excite PSPP [58,137,138,139,140,141,142]. The thin metal grating configuration coated on dielectric grating is one of the 1D metallic gratings [58,143]. Baeck et al. fabricated an Au-covered ${\mathrm{TiO}}_{2}$ gratings sensor utilizing soft-lithography-based templating technology [58]. By adjusting the thermal annealing process, the sensitivity of the proposed sensor was up to 920.45 nm/RIU. Two-dimensional gratings have also been observed to excite PSPP, and the surface relief metal grating configuration is one of them. Srijit et al. fabricated the crossed surface relief gratings biosensor (CSRGs) in Figure 7(a-1). The biosensor achieved the sensitivity of 647.8 nm/RIU in Figure 7(a-2) [59], which was three times higher than that of the simple surface relief gratings biosensor (188.35 nm/RIU) [140]. The study showed that using the 2D metallic grating can enhance the sensor performance. In addition, CSRGs were first used to monitor the biomolecule binding events. Streptavidin was detected by the streptavidin–biotin affinity model, and the LOD was 3.8 nM in Figure 7(a-3). CSRGs had the advantages of ease of fabrication and low cost (<10 cents/unit). However, the utilization of CSRGs in corrosive solutions had some limitations because azo-glass degraded when it interacted with strong solvents.

The grating-coupled metal-dielectric MHMM biosensors have been developed [53,54,60]. Kandammathe et al. fabricated a Au/${\mathrm{Al}}_{2}O_{3}$ MHMM biosensor, and a nanohole grating was placed on the top of the MHMM to provide extra wave vectors for the excitation of high-k modes in Figure 7(b-1). Both wavelength and angle scanning modes have been demonstrated for MHMM biosensors [53,54]. The MHMM biosensor can cover the visible and near-infrared spectral ranges, achieving a maximum sensitivity of 30,000 nm/RIU and a corresponding FOM of 590/RIU in the wavelength-scanning mode in Figure 7(b-2). The high sensitivity results from the coupling between the grating surface modes and high-k modes, which exhibit a strong dependence on the refractive index changes in the surrounding environment. In addition, biotins at a picomole concentration were detected by the biotin–streptavidin affinity model, shown in Figure 7(b-3). A higher sensitivity of 7000 deg/RIU was achieved in the angle scanning mode due to the higher signal-to-noise ratio, which was much higher than that of the PSPP biosensor [54,60]. The MHMM biosensor detected the Cowpea mosaic virus at concentrations as low as 1 fM. The miniaturization process of MHMM biosensors can currently achieve sizes as small as hundreds of nanometers using nanolithography technologies. However, the transverse size of these sensors is limited by the optical reading cross section, which typically ranges in the tens of microns. Nonetheless, the transverse cross section of the sensors was still within the range of several hundred nanometers. An overview of propagating plasmonic eigenwaves biosensors based on the grating-coupled mechanism is reported in Table A3.

### 4.3. Coupled Propagating-Localized Plasmonic Biosensors

We reviewed the localized and propagating plasmonic eigenwaves biosensors in Section 4.1 and Section 4.2, respectively. When PSPP and LSPR are excited by incident light, they can generate the evanescent field, and the penetration depth of the former is an order of magnitude longer than that of the latter. Moreover, LSPR has highly localized fields with greater enhancement. They each have their own distinct advantages.

To obtain the great penetration depth and the localized field enhancement simultaneously, several novel configurations are proposed, which allow us to couple PSPP to LSPR. The periodic nanostructure array can be fabricated on the thin metal film to achieve the co-excitation of both PSPP and LPSR when the array is treated as a grating to provide the additional wave vector [144,145]. In addition, the nanoholes array also achieves the simultaneous excitation of PSP and LSP [146,147,148,149,150,151,152,153]. To improve the sensitivity of the PSPP biosensors, the sandwich immunoassay approach was used. The detected molecules were sandwiched between the primary antibody-functionalized metal film and the secondary antibody-functionalized nanoparticles, in which the coupling effect between PSPP and LSPR played a dominant role. As the nanoparticles moved further away from the surface of the metal film, the coupling effect gradually weakened [154].

**Figure 8:**
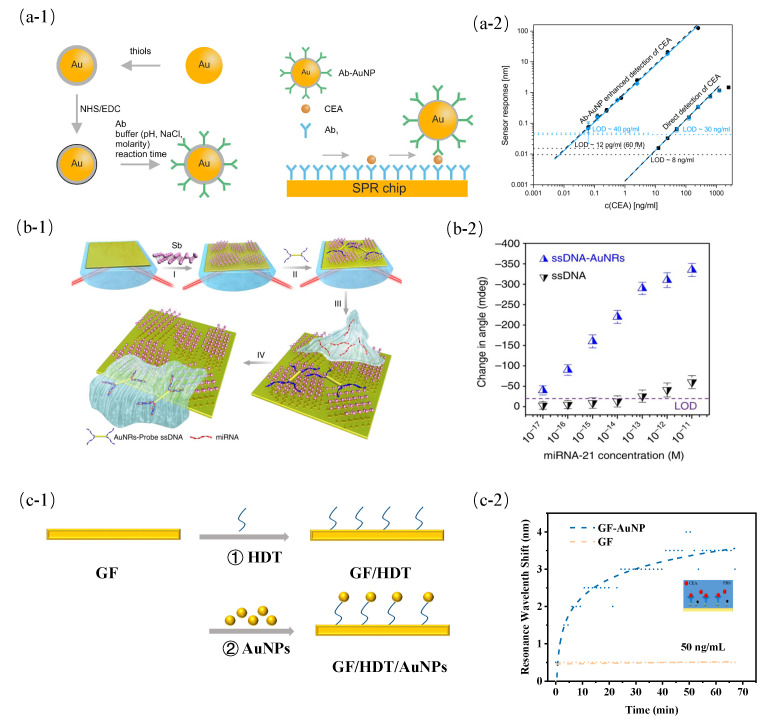
(**a-1**) (**Left panel**): Synthesis of the secondary antibody-functionalized gold nanospheres. (**Right panel**): Schematic of the sandwich immunoassay for CEA detection. (**a-2**) Fitting curves between the biosensor response and CEA concentrations for the sandwich immunoassay detection and the direct detection. Reproduced from Ref. [61] with permission. Copyright © 2017, Springer-Verlag Berlin Heidelberg. (**b-1**) Sandwich immunoassay schematic of a PSPP biosensor with AuNRs for miRNA detection. (**b-2**) Change in angle response to different miRNA-21 concentrations with or without AuNRs. Reproduced from Ref. [62] with permission. Copyright © 2019, the author(s). (**c-1**) Fabrication of GF-AuNPs. (**c-2**) Resonance wavelength shift with time of GF or GF-AuNPs biosensors for 50 ng/mL CEA. Reproduced from Ref. [64] with permission from the Royal Society of Chemistry.

The sandwich immunoassay has also been reported for PSPP sensors in the detection of carcinoembryonic antigen (CEA) [61,155,156]. Figure 8(a-1) shows the schematic of the sandwich immunoassay for CEA detection. By optimizing the preparation conditions on the secondary antibody functionalized gold nanoparticles, the response of the PSPP biosensor increased by 1000 times and detected CEA at concentrations of 40 pg/mL in the 50% blood plasma in Figure 8(a-2) [61]. A long-range PSPP (LRSPP) had longer penetration depth than conventional PSPP. By optimizing the size and density of gold nanoparticles, the LRSPP biosensors achieved up to 50-fold improvement in sensitivity. However, the use of the gold nanoparticles-based sandwich immunoassay methods proved to be more effective in conventional PSPP biosensors in comparison with LRSPR biosensors [157]. Tianyu et al. reported a similar approach with a few modifications [62]. The sandwich format was made of antimonene-modified gold film and ssDNA-functionalized gold nanorods (AuNRs) as shown in Figure 8(b-1). The LOD of the proposed biosensor for miRNA was 10 aM, which was far better than many miRNA biosensors as shown in Figure 8(b-2). Wu et al. investigated the performances of biosensors coated by carboxyl-modified GO (COOH-GO) for pig IgG detection [63]. The antibody attached to biosensors was used to interact with the gold nanostar–antigen bioconjugates. It showed that COOH-GO-coated biosensors had more active sites and the LOD of the biosensor was 0.0375 μg/mL.

The sandwich immunoassay has complex biological modification steps. Gold nanoparticles directly modified by gold film can also achieve the coupling of PSPP to LSPR. Recently, a resonant coupling biosensor between PSPP and LSPR supported by gold nanospheres was fabricated, and the influence of evanescent field intensity and distribution on sensitivity was investigated [64]. The nanosphere-modified gold film (GF-AuNPs) was fabricated by the combination between electron beam evaporation technology and chemical reactions in Figure 8(c-1). The PSPP was excited under the prism-coupling mechanism, and then it stimulated LSPR supported by nanospheres, which resulted in the resonant coupling between PSPP and LSPR. In comparison with PSPP, the surface electric field of the resonant coupling mode increased by 4.6 times, and the sensing performance had 7-fold improvement in the CEA detection in Figure 8(c-2).

## 5. Plasmon Nanorulers Biosensing

The plasmon nanorulers can be used to detect molecules in the length range of 1–100 nm [158]. The sensing principle of plasmon nanorulers biosensors is measuring the shifts in the resonance peaks or observing the color changes, which are associated with the detected length. The LSPR peaks indicate the maximum absorption and scattering of photons at specific wavelengths. The detected molecules can change the distance between the nanoparticles, causing an overall response change, such as color changes. Variations in distance between nanostructures can influence the mode coupling effect, resulting in spectra shifts. When the detected molecules change the distance between nanostructures, the molecules can be detected. Plasmon nanorulers biosensors are classified into 2D plasmon nanorulers biosensors in Section 5.1 and three-dimensional (3D) plasmon nanorulers sensors in Section 5.2 according to the detection dimension.

### 5.1. 2D Plasmon Nanoruler Biosensors

The vibrant colors of the gold nanoparticles colloidal solution have made them a subject of great interest among physicists and chemists [159], and are also widely used in various fields of biology and medicine. Additionally, metal nanoparticles like silver, aluminum, and copper exhibit plasmonic responses in the ultraviolet or visible regions [160]. In terms of the chemical stability, biocompatibility, and ease of functionalization, they may not have the same level. When two gold nanoparticles come into close contact, the fields resulting from LSPR are coupled to each other, and the resonant peak shifts to longer wavelengths [161,162]. The impact of a nearby active species on the LSPR frequency is often greater than that of a change in the refractive index [163]. As a result, many biosensing studies have utilized plasmonic coupling to generate substantial changes in the detected signal.

Without stabilization factors like repulsion from net charge or steric hindrance caused by substances on the surface of nanoparticles, the van der Waals forces between the nanoparticles can lead to their aggregation. Then, the color of the gold nanoparticles solution turns blue or purple [164], which is the main mechanism for achieving a visible readout with naked eyes. This aggregation can be initiated by the binding of molecules or can result from a cascade of reactions that occur following molecular recognition. The color changes of the solution are quite remarkable.

**Figure 9:**
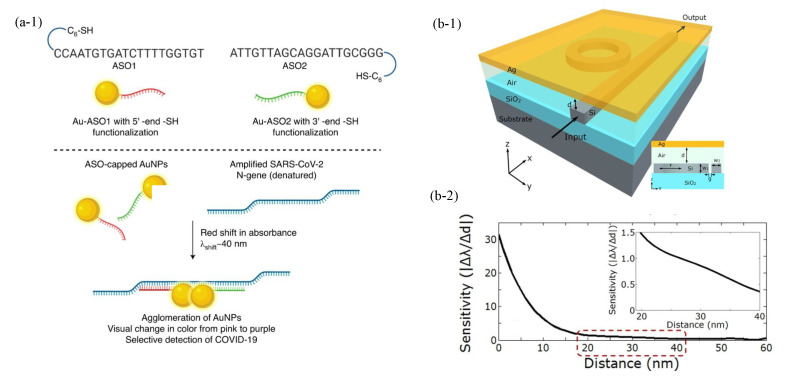
(**a-1**) Schematic of the specific binding of two nanosoheres and the composition of the plasmon nanoruler. Reproduced from Ref. [78] with permission. Copyright © 2021, the author(s), under exclusive license to Springer Nature Limited. (**b-1**) Schematic of hybrid plasmonic microring nanoruler comprised of a microring resonator, a space layer, and a silver film. (**b-2**) Sensitivity as a function of distance between the microring resonator and the silver film. Reproduced from Ref. [72] with permission. Copyright © 2018, The Author(s).

The plasmon nanorulers can be composed of two gold nanoparticles, which are brought very close together by the specific recognition of two nucleic acid sequences [19,75,76,77,78,79,80,81]. Gold nanoparticles can be functionalized by thiol-modified sequences that are complementary to the target nucleic acid sequences. In a basic design, two sets of sequences-modified gold nanoparticles are synthesized and hybridized with two different adjacent sequences on the target nucleic acid. When the target nucleic acid is absent, the color of the sequences’ modified gold nanoparticles solution is bright red. However, in the presence of target nucleic acid and at the right temperature, the gold nanoparticles will aggregate, and the solution color will change from red to blue. Ginger and colleagues successfully detected DNA hybridization in up to 50% serum [65]. Figure 9(a-1) shows the schematic of the specific binding of two nanosoheres by the target DNA. This method has been also used to detect RNA viruses like SARS-CoV-2, with a reported LOD of 10 copies of target RNA per μL of clinical samples [78]. In addition, the accuracy and specificity of the detection were found to be >98.4% and 100%, respectively. An alternative method for identifying specific nucleic acid sequences targets involves the utilization of DNAzymes. These DNAzymes disconnect a sequence linker that binds gold nanoparticles together, resulting in their dispersion, and the solution color is changed to red [158,165]. The quality of gold nanoparticles is influenced by the concentration of hydrogen peroxide when they are synthesized through a hydrogen peroxide-mediated reduction of gold ions that are added to the test solution [66,166]. When the concentration is high (or low), the color of the gold nanoparticles solution is red (or blue). Based on the principle illustrated above, the prostate-specific antigen detected the LOD of 1 × $10^{-18}$ g/mL [66]. The mechanism of the color transformation of gold nanoparticles mentioned above can also be used for the detection of ions or small molecules [67,68,69,70]. However, there are still some challenges to be addressed in the plasmon nanoruler of colloidal systems, including stability issues, detecting samples with high ionic strength, and the lack of integration into a simple platform.

Plasmon nanorulers can be also composed of the dimer model, which consists of two nanoparticles and a linker tethered between them. In the dimer model, the incident light can excite the longitudinal and vertical plasmon modes. These modes are characterized by the head-to-tail or parallel alignment of electric field directions. The resonance frequency of the modes changes when the distance between the nanoparticles decreases. Specifically, the longitudinal mode experiences a redshift, while the vertical mode undergoes a blueshift. This distance-dependent scattering peak provides information about the precise distance of the dimer nanostructures. The sensing principle of the dimer model is based on measuring the shift of the resonance peaks. The distance between two gold nanoparticles can be altered by introducing the detected molecules, such as a protein, leading to a significant change in the plasmonic resonance wavelength. The dark-field scattering spectra can be used to record the properties of the detected molecules. For example, Ye et al. conducted a study on the monitoring of the conformational dynamics of a heat shock protein 90 (Hsp90) using plasmon nanorulers comprising two gold nanospheres [71]. A glass slide was utilized to immobilize one gold nanosphere as an anchor, while another gold nanosphere was attached to the anchor nanosphere via an Hsp90 protein. The alteration in the interparticle distance caused a shift in the LSPR peak, facilitating the differentiation between the open and closed conformations of the Hsp90 protein connecting the two gold nanospheres. A plasmon nanoruler, consisting of pairs of nanoparticles, can accurately analyze the characteristics of a single biological molecule.

In addition, the stereo structures can also achieve plasmon nanorulers biosensors. For example, PSPP was applied in 1D plasmon nanorulers to promote the development of the sensing field [72]. By combining the concepts of the PSPP, resonators, and waveguides, a novel hybrid plasmonic microring nanoruler was proposed. As illustrated in Figure 9(b-1), the stereo structure of the hybrid plasmonic microring nanoruler is composed of a microring resonator, a suspending silver layer, and the gap region between them is air. The hybrid mode can be considered a supermode resulting from the combination of PSPP and the discontinuity of the electric field at the interface between silicon and air. The changes in the distance between the silicon waveguide and the silver layer can lead to changes in the effective mode index, which can cause the resonance peak shifts of the spectra at the output side of the silicon waveguide. The sensitivity of the system was observed to increase rapidly as the distance decreased as shown in Figure 9(b-2). The sensitivity exceeded 14.8 nm/nm with the distance less than 5 nm. Nan et al. proposed a stereo plasmon nanoruler biosensor, which was composed of a suspending gold nanohole array layer, a flat gold film, and a gap region between them [73]. When the thickness of the gap region decreased, the characteristic reflection dips were redshifted, which resulted from the enhancement of the coupling effect between the gold nanohole layer and the gold layer. The coupling efficiency was substantially increased by highly confined surface charges resulting from Bloch wave surface plasmon polarizations. Based on the sensing principle, the plasmon nanoruler biosensor achieved a thickness sensitivity of up to 61 nm/nm. The label-free quantification of procalcitonin was realized, and a very low LOD of 11.9 pg/mL was detected for clinical serum samples. This study provides new insights into the design of the stereo plasmon nanorulers biosensors. However, the application of plasmon nanorulers to POC diagnosis still needs to be explored due to the complex operational steps associated with biomolecules [19,65,167,168,169]. An overview of the recently reported plasmon nanoruler biosensors is reported in Table A4.

### 5.2. 3D Plasmon Nanoruler Sensors

**Figure 10:**
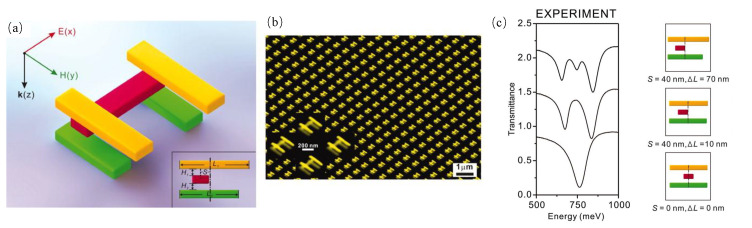
(**a**) Schematic of a 3D plasmon nanoruler biosensor of the ‘H−like’ structure. (**b**) SEM image of the 3D plasmon nanoruler biosensor. (**c**) Experimental transmittance spectra of the 3D plasmon nanoruler biosensor when the position of the nanorods changed. Reproduced from Ref. [74] with permission. Copyright © 2011, The American Association for the Advancement of Science.

While the 2D plasmon nanoruler biosensors are capable of investigating the fundamental parameters of molecules at the subwavelength scales, they do not provide a comprehensive understanding of motion in soft matter. As illustrated in Figure 10a,b, a 3D plasmon nanoruler of the ‘H−like’ structure was fabricated by EBL and layer-by-layer nano-stacking techniques [74]. The length and direction of individual nanorods were controlled. When the position of the middle gold nanorod changes, the quadrupole mode with distinct plasmon resonance is observed as shown in Figure 10c. The scattering spectrum showed high sensitivity when the configuration of the 3D plasmon nanoruler changed. The incident light can induce the dipolar resonance, which can be greatly influenced by incident electromagnetic radiation. When two nanorods were positioned parallel to each other, there were specific conditions, where quadrupolar coupling with the incident radiation took place. This led to a more pronounced resonance, as the radiative damping was significantly suppressed. Slight changes in the structure of the 3D plasmon nanoruler can generate an observable optical signal, enabling high-resolution sensing. The authors also proposed that the biomolecules can be used to connect the 3D plasmon nanoruler for biomolecule detection. For instance, the macromolecules, such as DNA/RNA or proteins, can connect to the various positions of the 3D plasmon nanoruler. Subsequently, the scattering spectrum can be measured using dark-field microscopy to determine the conformational structures of the target biomolecules. The nanolithography steps required for this process are highly intricate. Overcoming the challenge of using nanoparticles and biochemical linkers to achieve a 3D plasmon nanoruler is a significant feat.

Plasmon nanoruler biosensors continue to be a subject of ongoing research, with the expectation that future advancements will lead to constructs displaying even higher levels of sensitivity and selectivity. A breakthrough involves the development of a 3D plasmon nanoruler sensor, which exhibits remarkable sensitivity not only to the distance between nanoparticles but also to their spatial arrangement [74]. The plasmon nanoruler biosensors pave the way for a new generation of highly sensitive plasmonic biosensors capable of detecting minute changes in their surroundings.

## 6. SERS Biosensing

The Raman spectroscopy technique utilizes the Raman scattering light of a sample molecule to extract information about the composition and concentration. The positions of the characteristic peaks are determined by the vibrational frequency of each functional group, which is commonly referred to as the fingerprint nature of the Raman spectrum. To date, Raman spectroscopy has been successfully applied to the qualitative and quantitative determination of complex samples due to the advantages of the fingerprint spectroscopy, strong anti-interference ability, simple sample preparation, wide measurable spectral range, and being unaffected by solvent water [170,171,172]. However, the Raman scattering light signal is very weak, and it is difficult to distinguish from the fluorescent background, which is a major limitation in the application. SERS overcame this drawback and brought Raman spectroscopy back to life for applications.

The electromagnetic enhancement process can be comprehended through a two-step enhancement process. The initial step is attributed to the amplification of the local electromagnetic field around the plasmonic nanostructure at the incident light frequency $\omega_{0}$. This phenomenon can be mathematically represented as $E_{loc}\left(\omega_{0}\right)=G_{1}E_{0}$, where $G_{1}$ denotes the enhancement factor of the local electromagnetic field, and $E_{0}$ and $E_{loc}$ are the local electric fields in the absence and presence of nanoparticles, respectively. In the subsequent step, the plasmonic nanostructure acts as an optical antenna, facilitating the transmission of the Raman signal from the near field to the far field. The intensity of the Raman signal is directly proportional to the enhanced electromagnetic field of the Raman scattering at the corresponding Raman scattering frequency $\omega_{R}$. The local electromagnetic field is $E_{loc}\left(\omega_{R}\right)=G_{2}E_{0}$. Optimal SERS enhancement requires a delicate balance between the plasmon peaks of the metal nanostructures and the excitation and emission wavelengths. When the incident light frequency is close to the Raman scattering light frequency, there is $G_{1}$ = $G_{2}$. Therefore, the SERS enhancement factor can expressed as $G_{\mathrm{SERS}}=G_{1}^{2}G_{2}^{2}={\left|\frac{E_{loc}\left(\omega_{0}\right)}{E_{0}\left(\omega_{0}\right)}\right|}^{2}{\left|\frac{E_{loc}\left(\omega_{R}\right)}{E_{0}\left(\omega_{R}\right)}\right|}^{2}\approx {\left|\frac{E_{loc}\left(\omega_{R}\right)}{E_{0}\left(\omega_{0}\right)}\right|}^{4}$ [173].

In order to measure $EF_{\mathrm{SERS}}$ in experiments, several definitions can be used, including substrate and analytical enhancement factors [174]. However, in the platform-based biosensing, the commonly adopted definition is the substrate (or average) enhancement factor. It can be expressed as $EF_{\mathrm{SERS}}=\frac{I_{\mathrm{SERS}}}{I_{\mathrm{Raman}}}\frac{N_{\mathrm{Raman}}}{N_{\mathrm{SERS}}}$, where $I_{\mathrm{Raman}}$ and $I_{\mathrm{SERS}}$ represent the intensities of Raman and SERS signals, respectively, and $N_{\mathrm{Raman}}$ and $N_{\mathrm{SERS}}$ represent the average number of adsorbed molecules in the scattering volume for Raman and SERS measurements, respectively.

### 6.1. Metal Nanoparticle-Based SERS Biosensors

**Figure 11:**
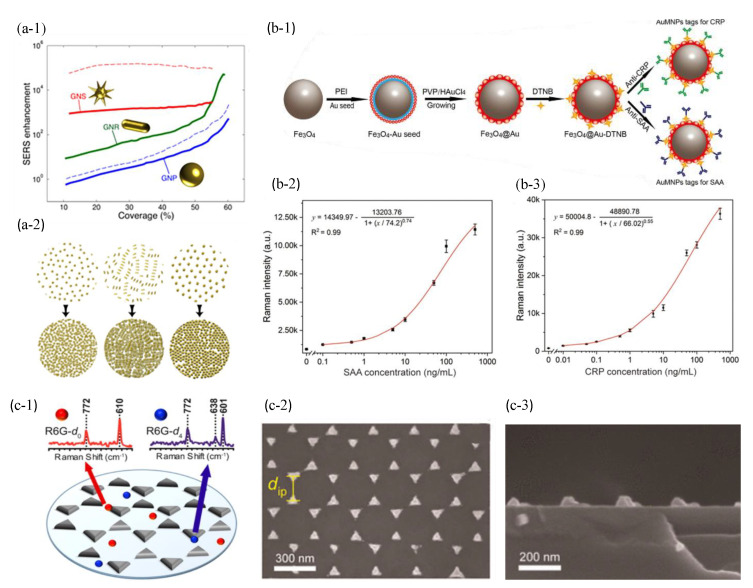
(**a-1**) Maximum SERS enhancement as a function of the coverage. The solid lines represented the incident light of 785 nm. The blue and red dashed line represented the incident lights of 633 nm and 900 nm for only nanospheres and only nanostars, respectively. (**a-2**) Schematic of the increased coverage of in the monolayers. Reproduced from Ref. [175] with permission. Under a Creative Commons license. (**b-1**) Fabrication schematic of antibody-conjugated Fe_3_O_4_@Au SERS nanotags. (**b-2**) Calibration curve between Raman intensity and SAA. (**b-3**) Calibration curve between Raman intensity and CRP. Reproduced from Ref. [86] with permission. Copyright © 2020 Elsevier B.V. (**c-1**) Schematic of the Ag triangular nanopyramids SERS subatrate. (**c-2**,**c-3**) SEM images of the Ag triangular nanopyramids in a top-down view and a cross-sectional view, respectively. Reproduced from Ref. [89] with permission. Copyright © 2013 American Chemical Society.

The strength of the SERS signal is highly dependent on the interfacial interactions between the molecules to be measured and the plasmonic nanostructure. Therefore, the maximum number of hot spots and the enhanced interaction of the target molecule with effective hot spots are two key parameters that determine the biosensing sensitivity. The sharp edges of nanostructures can significantly enhance the Raman scattering intensity. Solis et al. [175] demonstrated that the SERS enhancement of gold nanostars was far superior than that of nanospheres and nanorods in Figure 11(a-1). On the other hand, the nanoparticle morphologies that provided large electromagnetic enhancement at the single-particle level, such as nanostars, were not necessarily improved in closely aligned arrays. In contrast, when their surface density approaches complete coverage, simpler morphologies (e.g., spheres or rods) can significantly increase SERS enhancement as shown in Figure 11(a-1,a-2). Remarkably, plasmon-coupled nanorods, which have a surface coverage surpassing 60%, exhibited enhanced performance when compared to decoupled nanostars. This finding is illustrated in Figure 11(a-1). Since they perform two orders of magnitude better than nanosphere arrays and have similar, and occasionally better, performance compared to nanostars, nanorods were attractive candidates for the production of efficient SERS substrates. For each excitation wavelength and Raman shift, the shape of the nanoparticles can be adjusted to give the best electromagnetic enhancement.

Compared to solid nanoparticles, core-shell nanoparticles have less cytotoxicity, better dispersion, biocompatibility, and stability. In addition, core-shell nanostructures provide better near-field enhancement and lower LOD than the single nanoparticle. Huang et al. [88] used spiky Au@Ag nanoparticles to enhance the SERS signal by about 50 times compared with solid gold nanoparticles. Based on the linear fit plot depicting the peak intensity shift (559/cm)in relation to the logarithmic concentration of thiram, the LOD of thiram was determined to be 70 nM. In addition to the morphological differences, the thickness of the shell layer also had an effect on the SERS performance. The arrangement or positioning of metal atoms in the nanostructure can significantly impact fluctuations in SERS intensity. When the metal nanostructures are randomly distributed, it leads to inadequate SERS enhancement and non-reproducible hot spots. Wang et al. [176] investigated the effect of the shell layer thickness on the SERS enhancement. They synthesized Au@Ag core-shell nanoparticles through a chemical synthesis method. The LOD of 10^−12^ M R6G were able to be achieved at a shell thickness of about 8.5 nm. Special structures can be designed to further enhance the effective interaction between the target molecules and the hot spots to obtain lower LOD. However, it remains challenging for the detection of clinical samples with complex environmental factors (e.g., PH and salt ions) because it has an adverse effect on the stability of the colloidal solution, that is, gaps in colloidal system. A SERS-based lateral flow immunoassay strip [86] was developed incorporating functionalized Fe_3_O_4_@Au magnetic nanoparticles. The purpose of this strip was to achieve ultrasensitive and simultaneous analysis of dual infection biomarkers as shown in Figure 11(b-1). The SERS nanotags and efficient enrichment tools were created using Fe_3_O_4_@Au magnetic nanoparticles modified with antibodies. The Fe_3_O_4_@Au nanotags enabled the rapid capture and enrichment of target infection biomarkers from blood samples. The *LOD*s of serum amyloid A (SAA) and C-reactive protein (CRP) were determined to be 0.1 ng/mL and 0.01 ng/mL, respectively, as demonstrated in Figure 11(b-2,b-3). The magnetic SERS strips showed excellent stability, specificity and selectivity for the analysis of complex samples and show great potential in the field of infectious disease diagnosis.

The coupling of local plasmons between adjacent metal nanoparticles mostly generates hot spots that are typically less than 10 nm in size [177,178], and also plays a crucial role in enhancing the Raman scattering signal intensity. A dipolar coupling model can describe the electromagnetic interaction between adjacent local plasmons in the quasi-static limit [179,180]. As the interparticle spacing decreases to a very small value, the electrostatic interaction between nanoparticles intensifies. This interaction results in the formation of surface charge distributions that are highly nonsymmetrical. These distributions cause a more significant shift in the resonant dipolar oscillation and give rise to the emergence of higher multipolar modes [181,182]. Numerous studies have examined the presence of multipolar surface plasmon modes in nanoparticles and their impact on enhancing Raman scattering signals [92,93,94,95,96]. For example, SERS substrates were fabricated by growing Ag-nanoparticle arrays in anodized aluminum (AAO) nanochannels [93]. The influences of multipolar resonances on the SERS signal and the background noise were investigated. The second-order electric-quadrupole mode showed a stronger intensity of hot spots, which were confined to nanogaps between nanoparticles in comparison with the dipolar resonance. These hot spots can enhance the interaction between SERS substrates and analyte molecules. A significant reduction in SERS background was observed for these higher-order modes, as they had a small penetration depth inside the particles. This work provided potential design guidelines for SERS substrates with lower LOD.

### 6.2. Plasmonic Template-Based SERS Biosensors

**Figure 12:**
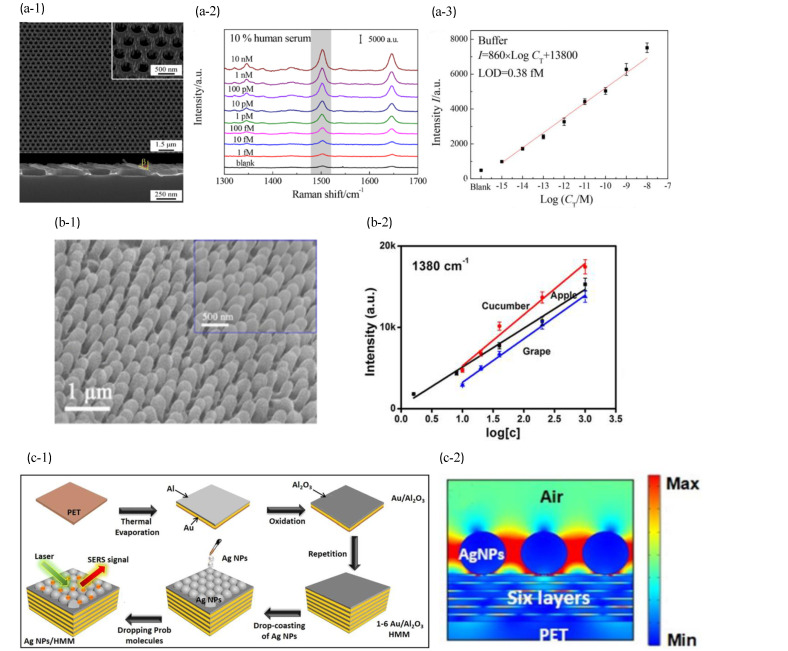
(**a-1**) SEM image of the Ag NR–NH array in a top-down view (**top**) and in a cross-sectional view (**bottom**). The inset is a magnified image. (**a-2**) SERS spectra of the target DNA at the concentrations ranging from $10^{-18}$ M to $10^{-15}$ M in the 10% human serum samples. (**a-3**) Semi-log plot between the SERS peak intensity and $C_{T}$ for the 1500/cm mode. Reproduced from Ref. [87] with permission. Copyright © 2020 American Chemical Society. (**b-1**) SEM image of the PDMS nano “tentacle” array. (**b-2**) Linear calibration curves between the SERS peak intensity and thiram concentration (red, cucumber; blue, grape; black, apple) for the 1380/cm mode. Reproduced from Ref. [183] with permission. Copyright © 2017 American Chemical Society. (**c-1**) Steps for preparing the Ag NPs/HMM SERS substrate. (**c-2**) The electric field simulation of the Ag NPs/HMM SERS substrate. Reproduced from Ref. [90] with permission. Copyright © 2021 Elsevier B.V.

A uniform array of nanostructured SERS substrates has a better enhancement factor and signal uniformity. Zrimsek et al. [89] prepared discrete Ag triangular nanopyramids by nanosphere lithography (NSL) and performed single-molecule detection. The simple fabrication procedure of NSL yielded large active arrays, shown in Figure 11(c-2,c-3). The enhanced electromagnetic fields were concentrated on the tips of individual triangular nanoparticle with ${EF}_{\mathrm{SERS}}$ as high as 10^8^ in Figure 11(c-1). Song et al. [87] developed a SERS biosensor by fabricating an array of silver nanorod-covered Ag nanoholes (Ag NR-NH) using a technology combination of NSL, reactive ion etching, oblique angle deposition, and physical evaporation deposition. The biosensor demonstrated highly sensitive detection of nucleic acids using a unique signal amplification strategy known as a DNA super sandwich as presented in Figure 12(a-1). The Ag NR-NH array, characterized by a large surface area and a uniformly arrayed nanostructure, exhibited remarkable anisotropic extraordinary optical transmission and strong LSPR. These properties enabled a sensitive plasmonic response to changes in the local refractive index and generated strong localized electric fields, resulting in excellent SERS activity. When detecting the target DNA in 50% human serum, the ${EF}_{\mathrm{SERS}}$ of the Ag NR-NH was found to be 4.02 × 10^6^, with a corresponding LOD of 0.77 fM as illustrated in Figure 12(a-2,a-3). Although NSL results in relatively large patches of organized structure, it is significant to note that grain boundary defects are also frequently observed. As the size of the beads decreases, the process of long-term sequencing becomes increasingly challenging.

Other array nanostructure can also result in similarly high ${EF}_{\mathrm{SERS}}$. A nano “tentacle” array SERS substrate was created and modeled like a gecko foot [183]. By employing AAO as a template and using polydimethylsiloxane to modify it, the substrate was created by the in situ deposition of Ag with hundreds of millions of highly flexible “tentacle” on its surface in Figure 12(b-1). The Ag nanoparticles, which can contact tiny areas of the sample surface in situ and achieve the in situ non-damaging enrichment of molecules to be measured in surface micro-regions, avoided sample pre-treatment. The Ag nanoparticles can generate rich hot spots among each other, which improves the detection sensitivity. The LOD of thiram in fruits and vegetables was up to 1.6 ng/cm^2^ as shown in Figure 12(b-2). Dan et al. [83] reported the preparation of novel nanorod bundles and their SERS performance with the substrate that detected R6G down to ∼10^−16^ M. Shafi et al. [90] successfully prepared a multilayer Ag nanoparticles HMM SERS substrate consisting of AgNPs and Au-Al_2_O_3_ membranes with enhanced SERS activity, shown in Figure 12(c-1). This study excited highly restricted BPP in the HMM in Figure 12(c-2). Experimental results showed that adding a multilayer stack of HMM could enhance the SERS signals, and the performance remained unchanged after six layers. Additionally, the SERS properties were affected by changes in the AgNPs gap at the top of the HMM. There was a maximum $EF_{\mathrm{SERS}}$ of 1.72 × 10^8^ when the gap was 10 nm. The excellent SERS sensitivity of the HMM substrate for detecting R6G solution has an LOD of 10^−12^ M. An overview of the recently reported SERS biosensors is reported in Table A5.

## 7. Advanced Application

Single-molecule sensing is achievable with the fast development of many kinds of remarkable biosensors and provides powerful tools for a broad range of biomedical research [184,185]. The single-molecule detection within complex mixtures is essential for diagnosis at an early stage, including cancer [186,187]. Among the detection techniques, the plasmonic biosensors have attracted extensive attention due to their high sensitivity, no pretreatment, cost effectiveness and time-saving nature. The implementation of an optical read-out scheme can be achieved by single-molecule SERS, and fluorescence.

### 7.1. Metal Nanoparticles Based Biosensors

The hot spots of the plasmonic biosensor boost the optical excitation and the interactions with the target molecule. SERS substrates serve as a good way to provide electric field enhancement, that is, more hot spots. Greater electric field enhancement can be achieved by adjusting the distance between the nanoparticles [188]. Highly ordered gold nanoparticles positioned at the tip of the silver wrinkled structure were fabricated, and the optimal plasma effect was achieved by stretching the PDMS substrate. A LOD of 10^−20^ M of CV and R6G molecules in water was achieved by the SERS effect. Postprocessing, such as the denoising algorithm and background reduction, can be conducted to further enhance the Raman signal [189]. Gold nanoparticles aggregation substrates connected with adenine were fabricated, and the plasmon enhancement of the stimulated Raman scattering with a low Raman cross section of 10^−30^ cm^2^ per molecule was achieved. Apart from the most studied Raman modes that parallel the probe tip, Rafael et al. [190] pointed out that the perpendicular components should also be considered, which could play an important role in the field enhancement. They used sodium chloride film to decouple the copper naphthalocyanine molecule from the Ag substrate. The tip-enhanced Raman spectroscopy images were measured and can be explained by analyzing the correlation between the three-dimensional components of the nanocavity and the original symmetry of the molecule.

The hot spots can also achieve significant enhancement of the fluorescence emission because the fluorescent molecules located nearby have enhanced absorption and emission characteristics [191]. For example, gold nanorods with length and width of 35 ± 2 and 17 ± 2 nm, respectively, were fabricated, and the plasmonic enhancement of fluorescence was used to detect the real-time binding of TmPyP4 to the GQs [192]. Multiple spectroscopies can also be achieved by simultaneously collecting the Raman scattering and the fluorescence spectra [193]. Both of the spectral signals were measured and correlated, and the real-time detection of bond cleavage reaction between xanthene and phenyl group was achieved. The electric field enhancement was up to about 80-fold with the Ag nanosphere and a plasmonic nanocavity substrate consisted of a 2 nm silica layer-coated Ag film.

### 7.2. Nanopores Based Biosensors

Nanopore biosensors consists of nanoscale apertures, where a single molecule can translocate in a one-dimensional condition [194]. The sensing signature of an unlabeled molecule can be achieved by the dwell time and event amplitude. Recently, the monolithic integration of the nanopore and plasmonic nanostructures provided a significant improvement in the single-molecule sensing performance [195]. The problem of the delivery of molecules in plasmonic sensors was solved, as they were confined to the nanopores. Moreover, the hot spots were highly overlapped with the tested molecules as they traversed through the pore, which resulted in a better signal-to-noise ratio. In this context, the optical means offered low noise, far-field measurement, fast detection and the possibility of multiplexing. Researchers have conducted experiments based on different nanostructures, such as nanorods [196], nanotriangles [197], inverted bowties [198], conical nanopores [199], and nanoslits [200].

**Figure 13:**
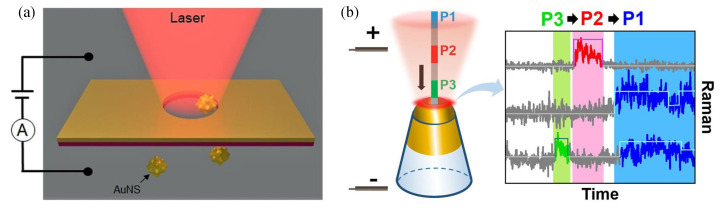
(**a**) Schematic diagram of the nanopore structure with 785 nm laser excitation. A single AuNS was trapped at the sidewall of the nanopore. Reproduced from Ref. [201] with permission. Copyright © 2020 Wiley-VCH Verlag GmbH and Co. KGaA, Weinheim. (**b**) Schematic diagram of the conical gold nanopore with bias voltage. The left part was the Raman response versus time and P1–P3 represented different segments. Reproduced from Ref. [199] with permission. Copyright © 2023, American Chemical Society.

For biosensors based on SERS measurements, the nanopores extend the observation time to sub-millisecond by translocating the molecule one at a time. But it is still too short to achieve the reliable spectrum measurement of a single molecule. One of the promising ways is to apply optical gradient force. The trapping of gold nanostars (AuNS) for seconds was experimentally demonstrated by a 785 nm laser [201]. Figure 13a showed the working principle, where a bias voltage of 0–3 V was applied to force the negatively charged AuNSs to translocate through the nanopore. Then, a 785 nm laser was illuminated on the nanopore to generate an intense plasmonic force, which can drag and trap the AuNSs to 5 nm from the sidewall of the nanopore for tens of seconds. The measurements at the single-molecule level included up to 10 distinct variations of amino acids, both with and without aromatic rings. The multiple parameters measurement can also be achieved in the nanopore-based biosensors. Both the SERS spectrum and ion current were detected based on a gold plasmonic nanopore biosensor [202]. The conformation transition and translocation behaviors of the calmodulin were simultaneously monitored. The optical force was used to extend the dwell time to longer than the acquisition time of 300 ms. Applying bias voltage is another common and easy method to control the translocation speed of molecules [197,199]. Figure 13b shows the single-molecule detection of cytochrome c by SERS in a conical gold nanopore [199]. High bias voltage was used to unfold the cytochrome c and slow down the translocation, then different SERS traces were recorded.

For biosensors based on fluorescence measurements, extensive studies have been conducted on plasmonic platforms, and single-molecule fluorescence sensing with spectral multiplexing has been commercialized, such as four-color code for DNA sequencing [203]. A so-called zero mode waveguide (ZMW), which cannot be observed by an optical microscope due to the sub-wavelength size, was realized by drilling sub-10 nm holes on a transparent thin Si_3_N_4_ membrane. Single-molecule detection of DNA translocation was achieved with fluorescent background suppression and a ten-fold net enhancement of the fluorescence intensity [204]. The nanoscale energy transfer between fluorescent molecules with gold nanopores of 80 nm diameter was also studied [205]. A simplified model exhibited good consistency with the experimental measurements, which presented potential opportunities in the areas of sequencing and conducting flow-through experiments. Other metal materials were also studied, such as Al and Pd [206,207]. A ZMW with freestanding Pd on SiN membranes was fabricated. The Pd had many advantages, such as lower optical background, smaller grain size than Au and more chemical inertness than Al. Then, better background suppression and premature bleaching prevention were achieved when the molecule passed through the dark side to the illuminated side.

Plasmonic nanostructure biosensors have experienced great development in the field of single-molecule sequencing [191,194,195], yet significant progress is still foreseeable. The control of the translocation speed of tested molecules is widely studied, where different methods, such as optical force [201,202,208,209], thermal effects [210,211,212,213] and bias voltage [197,199,214] can be applied. Multiple parameter measurements and multiplexing have great potential in application but still have many hurdles left to surmount [195,202]. One beneficial way to improve the sensors’ performance could be the joining of different measurement methods, like the combination of plasmonic sensors and nanopore sensors.

## 8. Summary and Perspectives

This review summarized three kinds of biosensors, including inherent resonance-based biosensors, plasmon nanoruler biosensors, and SERS biosensors. Two classical inherent resonance-based biosensors are PSPP-based and LSPR-based biosensors, both of which work on the principle of sensitivity to the surrounding refractive index changes. The lengths of the evanescent field of PSPP and LSPR are around 200–300 nm [113] and 5–15 nm [112], respectively. Otherwise, LSPR has greater local electric field enhancement than PSPP. The sensitivity of the inherent resonance-based biosensors can be expressed as the integral of the electric field at the region of refractive index changes [215,216]. The greater evanescent field length and electric field enhancement are conducive to improving sensitivity. The resonant coupling biosensors of PSPP to LSPR are superior options for refractive index sensing.

The plasmon nanoruler can detect the distance or length. Gold nanoparticles in solution and DNA sequences to be tested can construct a simple 2D plasmon nanoruler biosensor. The dimers in the solid phase can also form the plasmon nanoruler biosensors to detect biomolecules. The LSPR coupling effect is influenced by the biomolecules, which can be manifested as solution color changes or spectral shifts. Plasmon nanoruler biosensors based on coupling between different modes can be achieved by designing the various plasmonic nanostructures. Furthermore, a 3D plasmon nanoruler biosensor was also achieved by the ‘H-like’ structure. It shows that plasmon nanorulers not only detect length but also show sensitivity to spatial alignment.

SERS biosensors work on the amplification of the Raman scattering signal of the molecules to be tested. SERS has the characteristics of fingerprint specificity and high sensitivity. The electric field enhancement is positively correlated with the SERS biosensors performance. SERS substrates based on the metal nanoparticles and the plasmonic template are constructed to obtain greater electric field enhancement. SERS substrates with core-shell nanostructures have greater electric field enhancement than solid nanostructures. SERS substrates with sharp edge nanostructures and uniform arrangements of nanostructures can provide greater electric field enhancement.

Plasmonic nanostructure biosensors have experienced great development in the field of single-molecule detection, such as fluorescence and SERS, yet significant progress is still foreseeable. One beneficial way to improve the sensors performance could be the joining of different methods, such as optical force and thermal effects. More binding sites are important for detecting biomolecules, which can be achieved by increasing the surface area of the nanostructures. Two-dimensional materials are a good choice. Both nanoparticles sprayed on a flat surface and a patterned nanostructure can be used. For the accuracy of the detection results, dual-mode biosensors, such as those capable of both refractive index detection and SRES, are gradually being developed. It will undoubtedly drive the further development of plasmonic nanostructure biosensors. It could also pave the way for point-of-care diagnosis.

## Data Availability

No new data were created or analyzed in this study. Data sharing is not applicable to this article.

## Appendix A

**Table A1:** An overview of the recently reported Fano resonance-based biosensors.

Nanostructure	*FWHM*	Sensitivity	*FOM* $\left({\mathrm{RIU}}^{-1}\right)$	LOD (Detected Analytes)
capped gold nanoslits array [43]	3.68 nm	926 nm/RIU	252	- (BSA/antiBSA)
capped gold nanoslits array [42]	-	-	-	$∼\;\;10^{-11}$ g/mL (LMP1)
optical disc-based metasurfaces [40]	∼10 nm	1475 nm/RIU	147	25 μg/mL (protein G) $9\times 10^{3}$ copies/μL (gamma ray SARS-CoV-2)
array of narrow slit and nanohole pairs [41]	∼13 nm	1200 nm/RIU	92	-
Au grating [38]	10 nm	-	-	- (IgG)
metal-insulator-metal [37]	0.09 deg	∼86.76 deg/RIU	964 ± 150	30 nM (Bovine Serum Albumin))
Split-trench [39]	10 nm	687 nm/RIU	-	10 pM (bovine serum albumin)
Nanocrosses [44]	200 nm	1000 nm/RIU	5	-
Nanocrosses [45]	∼9 nm	1015 nm/RIU	108	200 pM (cytochrome c) 15 ng/mL (alpha-fetoprotein)

**Table A2:** An overview on propagating plasmonic eigenwaves biosensors based on prism-coupled mechanism.

Nanostructure	Resonant Wavelength (nm)	Sensitivity	LOD (Detected Analytes)
gold film [217]	-	31,400 nm/RIU	-
gold or silver film/${\mathrm{SiO}}_{2}$ [47]	650	68 deg/RIU	57.2 nM (CT)
gold film/Si/HMM array [46]	1155	43,000 nm/RIU	-
gold film/gold nanoparticles decorated ${\mathrm{MoS}}_{2}$ [50]	-	-	0.5 fM (miRNA)
gold film/Si/${\mathrm{MoSe}}_{2}$ [124]	1024	$1.1\times 10^{7}$ deg/RIU	-
gold film/graphene [48]	-	-	285 nM (kanamycin residues)
gold film/(GO(+)/GO(-) pair) [128]	-	150.38 ± 0.314 deg/RIU	-
nanorods array [52]	1200	32,000 nm/RIU	300 nM (biotin)
nanaorod HMMs [9]	1200	41,600 nm/RIU	2.4 nM (streptavidin)
platinum-di-selenide/gold film [218]	-	140.35 deg/RIU	1.95 nM (COVID-19)
gold film [219]	-	-	0.5 pg/mL (Ebola virus)
${\mathrm{TiO}}_{2}$/Ag/${\mathrm{MoSe}}_{2}$/graphene [220]	633	194 deg/RIU	0.0001 nM (COVID-19 SARS-CoV-2)
gold film [221]	-	$1.07\times 10^{6}$ mV/RIU	0.08 pg/mL (COVID-19 spike antigen detection)
gold film/Dithiobis (Succinimidyl Undecanoate)/NH_2_rGO- PAMAM [222]	-	0.2576 deg/pM	0.08 pM (dengue virus type 2 E-proteins)
ZnO/gold film [223]	∼640	2825 nm/RIU	2% (alcohol)
${\mathrm{WS}}_{2}$/${\mathrm{PtSe}}_{2}$/silver film [224]	633	194 deg/RIU	-

**Table A3:** An overview on propagating plasmonic eigenwaves biosensors based on grating-coupled mechanism.

Nanostructure	Resonant Wavelength (nm)	Sensitivity	LOD (Detected Analytes)
Au-covered ${\mathrm{TiO}}_{2}$ gratings [58]	1060	900 nm/RIU	-
crossed surface relief gratings [59]	-	647.8 nm/RIU	8.3 nM (streptavidin)
Au grating/(Au/${\mathrm{Al}}_{2}O_{3}$ multilayer HMM) [53]	1250	30,000 nm/RIU	10 pM (biotin)
Au grating/(Au/${\mathrm{Al}}_{2}O_{3}$ multilayer HMM) [54]	1250	7000 deg/RIU	1 fM (cowpea mosaic virus)

**Table A4:** An overview of the recently reported plasmon nanoruler biosensors.

Nanostructure	Sensitivity (nm/nm)	LOD (Detected Analytes)
nanosphere dimer [65]	-	10 pM (target DNA)
nanosphere solution [78]	-	10 copies/μL (coronavirus)
nanoparticles solution [165]	-	50 pM (DNA)
silver film/space layer/microring resonator [72]	14.8	-
Au nanohole/space layer/gold film [73]	61	11.9 pg/mL (procalcitonin)

**Table A5:** An overview of the recently reported SERS biosensors.

Nanostructure	Excitation Wavelength (nm)	${\mathrm{EF}}_{\mathrm{SERS}}$	Wavenumber (/cm)	LOD (Detected Analytes)
Au-decorated Ag nanorod arrays [83]	785	-	-	0.1 fM (R6G)
Au nanostars [84]	532	$2.5\times 10^{3}$	1374.9	1.51 mg/L (CV)
AgNPs-modified graphene nanoribbons [85]	532	$4.3\times 10^{5}$	1576	-
Fe_3_O_4_@Au magnetic nanoparticles [86]	785	$2.62\times 10^{6}$	1331	0.01 ng/mL (CRP)
Ag NR-NH array [87]	633	$4.02\times 10^{6}$	1500	0.7 fM (DNA)
Spiky Au@Ag core-shell nanoparticles [88]	785	$6.7\times 10^{7}$	559	70 nM (Thiram)
Ag triangular nanopyramids [89]	532	$10^{8}$	610	-
AgNPs based on HMMs [90]	532	$1.72\times 10^{8}$	710	1 pM (R6G,CV)
Hexagonal gold nanoparticles array [91]	785	$5.9\times 10^{9}$	736	0.8 pM (IL-6)

## References

[1] Anker J.N., Hall W.P., Lyandres O., Shah N.C., Zhao J., Van Duyne R.P. (2008). Biosensing with plasmonic nanosensors. Nat. Mater..

[2] Akimov A., Mukherjee A., Yu C., Chang D., Zibrov A., Hemmer P., Park H., Lukin M. (2007). Generation of single optical plasmons in metallic nanowires coupled to quantum dots. Nature.

[3] Ferry V.E., Sweatlock L.A., Pacifici D., Atwater H.A. (2008). Plasmonic nanostructure design for efficient light coupling into solar cells. Nano Lett..

[4] Koller D., Hohenau A., Ditlbacher H., Galler N., Reil F., Aussenegg F., Leitner A., List E., Krenn J. (2008). Organic plasmon-emitting diode. Nat. Photonics.

[5] DiChristina M., Meyerson B.S. The Top 10 Emerging Technologies of 2018.

[6] Meja-Salazar J., Oliveira O.N. (2018). Plasmonic biosensing: Focus review. Chem. Rev..

[7] Špačková B., Wrobel P., Bocková M., Homola J. (2016). Optical biosensors based on plasmonic nanostructures: A review. Proc. IEEE.

[8] Sugumaran S., Jamlos M.F., Ahmad M.N., Bellan C.S., Schreurs D. (2018). Nanostructured materials with plasmonic nanobiosensors for early cancer detection: A past and future prospect. Biosens. Bioelectron..

[9] Yan R., Wang T., Yue X., Wang H., Zhang Y.H., Xu P., Wang L., Wang Y., Zhang J. (2022). Highly sensitive plasmonic nanorod hyperbolic metamaterial biosensor. Photonics Res..

[10] Gao M., Yang W., Wang Z., Lin S., Zhu J., Yang Z. (2020). Plasmonic resonance-linewidth shrinkage to boost biosensing. Photonics Res..

[11] Arcadio F., Zeni L., Montemurro D., Eramo C., Di Ronza S., Perri C., D’agostino G., Chiaretti G., Porto G., Cennamo N. (2021). Biochemical sensing exploiting plasmonic sensors based on gold nanogratings and polymer optical fibers. Photonics Res..

[12] Tran N.H.T., Trinh K.T.L., Lee J.H., Yoon W.J., Ju H. (2018). Reproducible Enhancement of Fluorescence by Bimetal Mediated Surface Plasmon Coupled Emission for Highly Sensitive Quantitative Diagnosis of Double-Stranded DNA. Small.

[13] Yousefi H., Ali M.M., Su H.M., Filipe C.D., Didar T.F. (2018). Sentinel wraps: Real-time monitoring of food contamination by printing DNAzyme probes on food packaging. ACS Nano.

[14] Min J., Sempionatto J.R., Teymourian H., Wang J., Gao W. (2021). Wearable electrochemical biosensors in North America. Biosens. Bioelectron..

[15] Gupta R., Raza N., Bhardwaj S.K., Vikrant K., Kim K.H., Bhardwaj N. (2021). Advances in nanomaterial-based electrochemical biosensors for the detection of microbial toxins, pathogenic bacteria in food matrices. J. Hazard. Mater..

[16] Willets K.A., Van Duyne R.P. (2007). Localized surface plasmon resonance spectroscopy and sensing. Annu. Rev. Phys. Chem..

[17] Hutter E., Fendler J.H. (2004). Exploitation of localized surface plasmon resonance. Adv. Mater..

[18] Lin J.S., Tian X.D., Li G., Zhang F.L., Wang Y., Li J.F. (2022). Advanced plasmonic technologies for multi-scale biomedical imaging. Chem. Soc. Rev..

[19] Sönnichsen C., Reinhard B.M., Liphardt J., Alivisatos A.P. (2005). A molecular ruler based on plasmon coupling of single gold and silver nanoparticles. Nat. Biotechnol..

[20] Liu G.L., Yin Y., Kunchakarra S., Mukherjee B., Gerion D., Jett S.D., Bear D.G., Gray J.W., Alivisatos A.P., Lee L.P. (2006). A nanoplasmonic molecular ruler for measuring nuclease activity and DNA footprinting. Nat. Nanotechnol..

[21] Stryer L. (1978). Fluorescence energy transfer as a spectroscopic ruler. Annu. Rev. Biochem..

[22] Fan J.A., Wu C., Bao K., Bao J., Bardhan R., Halas N.J., Manoharan V.N., Nordlander P., Shvets G., Capasso F. (2010). Self-assembled plasmonic nanoparticle clusters. Science.

[23] Hentschel M., Saliba M., Vogelgesang R., Giessen H., Alivisatos A.P., Liu N. (2010). Transition from isolated to collective modes in plasmonic oligomers. Nano Lett..

[24] Lassiter J.B., Sobhani H., Fan J.A., Kundu J., Capasso F., Nordlander P., Halas N.J. (2010). Fano resonances in plasmonic nanoclusters: Geometrical and chemical tunability. Nano Lett..

[25] Payton J.L., Morton S.M., Moore J.E., Jensen L. (2014). A hybrid atomistic electrodynamics–quantum mechanical approach for simulating surface-enhanced Raman scattering. Accounts Chem. Res..

[26] Tong L., Zhu T., Liu Z. (2011). Approaching the electromagnetic mechanism of surface-enhanced Raman scattering: From self-assembled arrays to individual gold nanoparticles. Chem. Soc. Rev..

[27] Jensen L., Aikens C.M., Schatz G.C. (2008). Electronic structure methods for studying surface-enhanced Raman scattering. Chem. Soc. Rev..

[28] Pilot R., Signorini R., Durante C., Orian L., Bhamidipati M., Fabris L. (2019). A review on surface-enhanced Raman scattering. Biosensors.

[29] Xu T., Geng Z. (2021). Strategies to improve performances of LSPR biosensing: Structure, materials, and interface modification. Biosens. Bioelectron..

[30] Khan A.U., Zhao S., Liu G. (2016). Key parameter controlling the sensitivity of plasmonic metal nanoparticles: Aspect ratio. J. Phys. Chem. C.

[31] Sun Y., Xia Y. (2002). Increased sensitivity of surface plasmon resonance of gold nanoshells compared to that of gold solid colloids in response to environmental changes. Anal. Chem..

[32] Mahmoud M.A., El-Sayed M.A. (2010). Gold nanoframes: Very high surface plasmon fields and excellent near-infrared sensors. J. Am. Chem. Soc..

[33] Zhang Y., Charles D.E., Ledwith D.M., Aherne D., Cunningham S., Voisin M., Blau W.J., Gun’ko Y.K., Kelly J.M., Brennan-Fournet M.E. (2014). Wash-free highly sensitive detection of C-reactive protein using gold derivatised triangular silver nanoplates. RSC Adv..

[34] Tian L., Liu K.K., Morrissey J.J., Gandra N., Kharasch E.D., Singamaneni S. (2014). Gold nanocages with built-in artificial antibodies for label-free plasmonic biosensing. J. Mater. Chem. B.

[35] Hu T., Lin Y., Yan J., Di J. (2013). Synthesis of hollow gold nanoparticles on the surface of indium tin oxide glass and their application for plasmonic biosensor. Spectrochim. Acta Part A Mol. Biomol. Spectrosc..

[36] Mahmoud M.A., El-Sayed M.A. (2013). Substrate effect on the plasmonic sensing ability of hollow nanoparticles of different shapes. J. Phys. Chem. B.

[37] Qin J., Zhang Y., Liang X., Liu C., Wang C., Kang T., Lu H., Zhang L., Zhou P., Wang X. (2017). Ultrahigh figure-of-merit in metal–insulator–metal magnetoplasmonic sensors using low loss magneto-optical oxide thin films. ACS Photonics.

[38] Xiao B., Kogo G., Rutherford G.N., Bahoura M. (2019). Plasmonic pixel biosensor based on grazing angle illumination and computational imaging. IEEE Sens. J..

[39] Yoo D., Barik A., de León-Pérez F., Mohr D.A., Pelton M., Martín-Moreno L., Oh S.H. (2021). Plasmonic split-trench resonator for trapping and sensing. ACS Nano.

[40] Ahmed R., Guimarães C.F., Wang J., Soto F., Karim A.H., Zhang Z., Reis R.L., Akin D., Paulmurugan R., Demirci U. (2022). Large-Scale functionalized metasurface-based SARS-CoV-2 detection and quantification. ACS Nano.

[41] Huang W.X., Guo J.J., Wang M.S., Zhao G.R. (2017). Sensor based on Fano resonances of plane metamaterial with narrow slits. Phys. Lett. A.

[42] Hsieh H.Y., Chang R., Huang Y.Y., Juan P.H., Tahara H., Lee K.Y., Tsai M.H., Wei P.K., Sheen H.J., Fan Y.J. (2022). Continuous polymerase chain reaction microfluidics integrated with a gold-capped nanoslit sensing chip for Epstein-Barr virus detection. Biosens. Bioelectron..

[43] Lee K.L., Huang J.B., Chang J.W., Wu S.H., Wei P.K. (2015). Ultrasensitive biosensors using enhanced Fano resonances in capped gold nanoslit arrays. Sci. Rep..

[44] Verellen N., Van Dorpe P., Huang C., Lodewijks K., Vandenbosch G.A., Lagae L., Moshchalkov V.V. (2011). Plasmon line shaping using nanocrosses for high sensitivity localized surface plasmon resonance sensing. Nano Lett..

[45] Shen Y., Zhou J., Liu T., Tao Y., Jiang R., Liu M., Xiao G., Zhu J., Zhou Z.K., Wang X. (2013). Plasmonic gold mushroom arrays with refractive index sensing figures of merit approaching the theoretical limit. Nat. Commun..

[46] Wang H., Wang T., Yan R., Yue X., Wang L., Wang Y., Zhang J., Wang J. (2022). Coupling plasmon-waveguide resonance and multiple plasma modes in hyperbolic metamaterials for high-performance sensing. Nanotechnology.

[47] Abbas A., Linman M.J., Cheng Q. (2011). Sensitivity comparison of surface plasmon resonance and plasmon-waveguide resonance biosensors. Sens. Actuators B Chem..

[48] Écija-Arenas Á., Kirchner E.M., Hirsch T., Fernández-Romero J.M. (2021). Development of an aptamer-based SPR-biosensor for the determination of kanamycin residues in foods. Anal. Chim. Acta.

[49] Nurrohman D.T., Wang Y.H., Chiu N.F. (2020). Exploring graphene and MoS2 chips based surface plasmon resonance biosensors for diagnostic applications. Front. Chem..

[50] Nie W., Wang Q., Yang X., Zhang H., Li Z., Gao L., Zheng Y., Liu X., Wang K. (2017). High sensitivity surface plasmon resonance biosensor for detection of microRNA based on gold nanoparticles-decorated molybdenum sulfide. Anal. Chim. Acta.

[51] Huo P., Zhang S., Liang Y., Lu Y., Xu T. (2019). Hyperbolic metamaterials and metasurfaces: Fundamentals and applications. Adv. Opt. Mater..

[52] Kabashin A.V., Evans P., Pastkovsky S., Hendren W., Wurtz G.A., Atkinson R., Pollard R., Podolskiy V.A., Zayats A.V. (2009). Plasmonic nanorod metamaterials for biosensing. Nat. Mater..

[53] Sreekanth K.V., Alapan Y., ElKabbash M., Ilker E., Hinczewski M., Gurkan U.A., De Luca A., Strangi G. (2016). Extreme sensitivity biosensing platform based on hyperbolic metamaterials. Nat. Mater..

[54] Sreekanth K.V., Alapan Y., ElKabbash M., Wen A.M., Ilker E., Hinczewski M., Gurkan U.A., Steinmetz N.F., Strangi G. (2016). Enhancing the angular sensitivity of plasmonic sensors using hyperbolic metamaterials. Adv. Opt. Mater..

[55] Jiang L., Zeng S., Xu Z., Ouyang Q., Zhang D.H., Chong P.H.J., Coquet P., He S., Yong K.T. (2017). Multifunctional hyperbolic nanogroove metasurface for submolecular detection. Small.

[56] Sreekanth K.V., Mahalakshmi P., Han S., Mani Rajan M.S., Choudhury P.K., Singh R. (2019). Brewster mode-enhanced sensing with hyperbolic metamaterial. Adv. Opt. Mater..

[57] Palermo G., Sreekanth K.V., Maccaferri N., Lio G.E., Nicoletta G., De Angelis F., Hinczewski M., Strangi G. (2020). Hyperbolic dispersion metasurfaces for molecular biosensing. Nanophotonics.

[58] Choi B., Dou X., Fang Y., Phillips B.M., Jiang P. (2016). Outstanding surface plasmon resonance performance enabled by templated oxide gratings. Phys. Chem. Chem. Phys..

[59] Nair S., Escobedo C., Sabat R.G. (2017). Crossed surface relief gratings as nanoplasmonic biosensors. ACS Sens..

[60] Sreekanth K.V., ElKabbash M., Alapan Y., Ilker E.I., Hinczewski M., Gurkan U.A., Strangi G. (2017). Hyperbolic metamaterials-based plasmonic biosensor for fluid biopsy with single molecule sensitivity. EPJ Appl. Metamat..

[61] Špringer T., Chadtová Song X., Ermini M.L., Lamačová J., Homola J. (2017). Functional gold nanoparticles for optical affinity biosensing. Anal. Bioanal. Chem..

[62] Xue T., Liang W., Li Y., Sun Y., Xiang Y., Zhang Y., Dai Z., Duo Y., Wu L., Qi K. (2019). Ultrasensitive detection of miRNA with an antimonene-based surface plasmon resonance sensor. Nat. Commun..

[63] Wu Q., Sun Y., Ma P., Zhang D., Li S., Wang X., Song D. (2016). Gold nanostar-enhanced surface plasmon resonance biosensor based on carboxyl-functionalized graphene oxide. Anal. Chim. Acta.

[64] Wang H., Wang T., Zhong S., Zhang J., Yan R., Xu P., Zhang Y.h., Yue X., Wang L., Wang Y. (2023). Sensitivity investigation of a biosensor with resonant coupling of propagating surface plasmons to localized surface plasmons in the near infrared region. Nanoscale.

[65] Chen J.I., Chen Y., Ginger D.S. (2010). Plasmonic nanoparticle dimers for optical sensing of DNA in complex media. J. Am. Chem. Soc..

[66] De La Rica R., Stevens M.M. (2012). Plasmonic ELISA for the ultrasensitive detection of disease biomarkers with the naked eye. Nat. Nanotechnol..

[67] Liu J., Lu Y. (2003). A colorimetric lead biosensor using DNAzyme-directed assembly of gold nanoparticles. J. Am. Chem. Soc..

[68] Lee J.S., Han M.S., Mirkin C.A. (2007). Colorimetric detection of mercuric ion (Hg^2+^) in aqueous media using DNA-functionalized gold nanoparticles. Angew. Chem. Int. Ed..

[69] Liu J., Lu Y. (2006). Preparation of aptamer-linked gold nanoparticle purple aggregates for colorimetric sensing of analytes. Nat. Protoc..

[70] Famulok M., Hartig J.S., Mayer G. (2007). Functional aptamers and aptazymes in biotechnology, diagnostics, and therapy. Chem. Rev..

[71] Ye W., Gotz M., Celiksoy S., Tuting L., Ratzke C., Prasad J., Ricken J., Wegner S.V., Ahijado-Guzman R., Hugel T. (2018). Conformational dynamics of a single protein monitored for 24 h at video rate. Nano Lett..

[72] Du J., Wang J. (2018). Hybrid plasmonic microring nano-ruler. Sci. Rep..

[73] Nan J., Zhu S., Ye S., Sun W., Yue Y., Tang X., Shi J., Xu X., Zhang J., Yang B. (2020). Ultrahigh-Sensitivity Sandwiched Plasmon Ruler for Label-Free Clinical Diagnosis. Adv. Mater..

[74] Liu N., Hentschel M., Weiss T., Alivisatos A.P., Giessen H. (2011). Three-dimensional plasmon rulers. Science.

[75] Reinhard B.M., Siu M., Agarwal H., Alivisatos A.P., Liphardt J. (2005). Calibration of dynamic molecular rulers based on plasmon coupling between gold nanoparticles. Nano Lett..

[76] Elghanian R., Storhoff J.J., Mucic R.C., Letsinger R.L., Mirkin C.A. (1997). Selective colorimetric detection of polynucleotides based on the distance-dependent optical properties of gold nanoparticles. Science.

[77] Moitra P., Alafeef M., Dighe K., Frieman M.B., Pan D. (2020). Selective naked-eye detection of SARS-CoV-2 mediated by N gene targeted antisense oligonucleotide capped plasmonic nanoparticles. ACS Nano.

[78] Alafeef M., Moitra P., Dighe K., Pan D. (2021). RNA-extraction-free nano-amplified colorimetric test for point-of-care clinical diagnosis of COVID-19. Nat. Protoc..

[79] Notomi T., Mori Y., Tomita N., Kanda H. (2015). Loop-mediated isothermal amplification (LAMP): Principle, features, and future prospects. J. Microbiol..

[80] Tan E., Wong J., Nguyen D., Zhang Y., Erwin B., Van Ness L.K., Baker S.M., Galas D.J., Niemz A. (2005). Isothermal DNA amplification coupled with DNA nanosphere-based colorimetric detection. Anal. Chem..

[81] Oliveira B.B., Veigas B., Baptista P.V. (2021). Isothermal amplification of nucleic acids: The race for the next “gold standard”. Front. Sens..

[82] Xiao S., Qin M., Duan J., Wu F., Liu T. (2022). Polarization-controlled dynamically switchable high-harmonic generation from all-dielectric metasurfaces governed by dual bound states in the continuum. Phys. Rev. B.

[83] Dan Y., Zhong C., Zhu H., Wang J. (2019). Highly ordered Au-decorated Ag nanorod arrays as an ultrasensitive and reusable substrate for surface enhanced Raman scattering. Colloids Surfaces A Physicochem. Eng. Asp..

[84] Bibikova O., Haas J., López-Lorente A.I., Popov A., Kinnunen M., Meglinski I., Mizaikoff B. (2017). Towards enhanced optical sensor performance: SEIRA and SERS with plasmonic nanostars. Analyst.

[85] Nong J., Tang L., Lan G., Luo P., Li Z., Huang D., Shen J., Wei W. (2021). Combined Visible Plasmons of Ag Nanoparticles and Infrared Plasmons of Graphene Nanoribbons for High-Performance Surface-Enhanced Raman and Infrared Spectroscopies. Small.

[86] Liu X., Yang X., Li K., Liu H., Xiao R., Wang W., Wang C., Wang S. (2020). Fe_3_O_4_@ Au SERS tags-based lateral flow assay for simultaneous detection of serum amyloid A and C-reactive protein in unprocessed blood sample. Sens. Actuators B Chem..

[87] Song C., Jiang X., Yang Y., Zhang J., Larson S., Zhao Y., Wang L. (2020). High-sensitive assay of nucleic acid using tetrahedral DNA probes and DNA concatamers with a surface-enhanced Raman scattering/surface plasmon resonance dual-mode biosensor based on a silver nanorod-covered silver nanohole array. ACS Appl. Mater. Interfaces.

[88] Huang Z., Meng G., Hu X., Pan Q., Huo D., Zhou H., Ke Y., Wu N. (2019). Plasmon-tunable Au@ Ag core-shell spiky nanoparticles for surface-enhanced Raman scattering. Nano Res..

[89] Zrimsek A.B., Henry A.I., Van Duyne R.P. (2013). Single molecule surface-enhanced Raman spectroscopy without nanogaps. J. Phys. Chem. Lett..

[90] Shafi M., Liu R., Zha Z., Li C., Du X., Wali S., Jiang S., Man B., Liu M. (2021). Highly efficient SERS substrates with different Ag interparticle nanogaps based on hyperbolic metamaterials. Appl. Surf. Sci..

[91] Muhammad M., Shao C.s., Huang Q. (2021). Aptamer-functionalized Au nanoparticles array as the effective SERS biosensor for label-free detection of interleukin-6 in serum. Sens. Actuators B Chem..

[92] Chang Y.C., Dvoynenko M.M., Ke H., Hsiao H.H., Wang Y.L., Wang J.K. (2021). Double Resonance SERS Substrates: Ag Nanoparticles on Grating. J. Phys. Chem. C.

[93] Hsiao H.H., Ke H., Dvoynenko M.M., Wang J.K. (2020). Multipolar resonances of Ag nanoparticle arrays in anodic aluminum oxide nanochannels for enhanced hot spot intensity and signal-to-background ratio in surface-enhanced raman scattering. ACS Appl. Nano Mater..

[94] Pandey P., Seo M.K., Shin K.H., Lee Y.W., Sohn J.I. (2022). Hierarchically Assembled Plasmonic Metal-Dielectric-Metal Hybrid Nano-Architectures for High-Sensitivity SERS Detection. Nanomaterials.

[95] D’Andrea C., Bochterle J., Toma A., Huck C., Neubrech F., Messina E., Fazio B., Marago O.M., Di Fabrizio E., Lamy de La Chapelle M. (2013). Optical nanoantennas for multiband surface-enhanced infrared and Raman spectroscopy. ACS Nano.

[96] Dawson P., Duenas J., Boyle M., Doherty M., Bell S., Kern A., Martin O., Teh A.S., Teo K., Milne W. (2011). Combined antenna and localized plasmon resonance in Raman scattering from random arrays of silver-coated, vertically aligned multiwalled carbon nanotubes. Nano Lett..

[97] Bohren C.F., Huffman D.R. (2008). Absorption and Scattering of Light by Small Particles.

[98] Jain P.K., Lee K.S., El-Sayed I.H., El-Sayed M.A. (2006). Calculated absorption and scattering properties of gold nanoparticles of different size, shape, and composition: Applications in biological imaging and biomedicine. J. Phys. Chem. B.

[99] Schasfoort R. (2017). Surface plasmon resonance instruments. Handbook of Surface Plasmon Resonance.

[100] Pitarke J., Silkin V., Chulkov E., Echenique P. (2006). Theory of surface plasmons and surface-plasmon polaritons. Rep. Prog. Phys..

[101] Zayats A.V., Smolyaninov I.I., Maradudin A.A. (2005). Nano-optics of surface plasmon polaritons. Phys. Rep..

[102] Homola J., Yee S.S., Gauglitz G. (1999). Surface plasmon resonance sensors. Sens. Actuators B Chem..

[103] Kretschmann E., Raether H. (1968). Plasma resonance emission in solid bodies(Plasma resonance light emission mechanisms in solid bodies, discussing production by electron and light irradiation of body surface). Z. Naturforschung Ausg. A.

[104] Wang H., Brandl D.W., Le F., Nordlander P., Halas N.J. (2006). Nanorice: A hybrid plasmonic nanostructure. Nano Lett..

[105] Sherry L.J., Chang S.H., Schatz G.C., Van Duyne R.P., Wiley B.J., Xia Y. (2005). Localized surface plasmon resonance spectroscopy of single silver nanocubes. Nano Lett..

[106] Bukasov R., Shumaker-Parry J.S. (2007). Highly tunable infrared extinction properties of gold nanocrescents. Nano Lett..

[107] Jia P., Kong D., Ebendorff-Heidepriem H. (2020). Flexible plasmonic tapes with nanohole and nanoparticle arrays for refractometric and strain sensing. ACS Appl. Nano Mater..

[108] Otte M.A., Sepulveda B., Ni W., Juste J.P., Liz-Marzán L.M., Lechuga L.M. (2010). Identification of the optimal spectral region for plasmonic and nanoplasmonic sensing. ACS Nano.

[109] Ozbay E. (2006). Plasmonics: Merging photonics and electronics at nanoscale dimensions. Science.

[110] Gramotnev D.K., Bozhevolnyi S.I. (2010). Plasmonics beyond the diffraction limit. Nat. Photonics.

[111] Zhang J., Wang T., Yan R., Wang H., Yue X., Wang L., Wang Y., Yuan X., Wang J. (2023). Design of self-coupled plasmonic hyperbolic metamaterials refractive index sensor based on intensity shift. Phys. Scr..

[112] Haes A.J., Zou S., Schatz G.C., Van Duyne R.P. (2004). A nanoscale optical biosensor: The long range distance dependence of the localized surface plasmon resonance of noble metal nanoparticles. J. Phys. Chem. B.

[113] Jung L.S., Campbell C.T., Chinowsky T.M., Mar M.N., Yee S.S. (1998). Quantitative interpretation of the response of surface plasmon resonance sensors to adsorbed films. Langmuir.

[114] Kim S., Yoon S. (2021). On the Origin of the Plasmonic Properties of Gold Nanoparticles. Bull. Korean Chem. Soc..

[115] Jazayeri M.H., Amani H., Pourfatollah A.A., Pazoki-Toroudi H., Sedighimoghaddam B. (2016). Various methods of gold nanoparticles (GNPs) conjugation to antibodies. Sens. Bio-Sens. Res..

[116] Ryu K.R., Ha J.W. (2021). Enhanced detection sensitivity of the chemisorption of pyridine and biotinylated proteins at localized surface plasmon resonance inflection points in single gold nanorods. Analyst.

[117] Chen Y., Xianyu Y., Jiang X. (2017). Surface modification of gold nanoparticles with small molecules for biochemical analysis. Acc. Chem. Res..

[118] Olson J., Dominguez-Medina S., Hoggard A., Wang L.Y., Chang W.S., Link S. (2015). Optical characterization of single plasmonic nanoparticles. Chem. Soc. Rev..

[119] Fan J., Cheng Y., Sun M. (2020). Functionalized gold nanoparticles: Synthesis, properties and biomedical applications. Chem. Rec..

[120] Gao P.F., Li Y.F., Huang C.Z. (2019). Localized surface plasmon resonance scattering imaging and spectroscopy for real-time reaction monitoring. Appl. Spectrosc. Rev..

[121] Xu Y., Bai P., Zhou X., Akimov Y., Png C.E., Ang L.K., Knoll W., Wu L. (2019). Optical refractive index sensors with plasmonic and photonic structures: Promising and inconvenient truth. Adv. Opt. Mater..

[122] Wang Y., Wang T., Yan R., Yue X., Wang L., Wang H., Zhang J., Yuan X., Zeng J., Wang J. (2023). A Tunable Strong Electric Field Ultra-Narrow-Band Fano Resonance Hybrid Metamaterial Sensor Based on LSPR. IEEE Sens. J..

[123] Wu L., Chu H.S., Koh W.S., Li E.P. (2010). Highly sensitive graphene biosensors based on surface plasmon resonance. Opt. Express.

[124] Ouyang Q., Zeng S., Jiang L., Qu J., Dinh X.Q., Qian J., He S., Coquet P., Yong K.T. (2017). Two-dimensional transition metal dichalcogenide enhanced phase-sensitive plasmonic biosensors: Theoretical insight. J. Phys. Chem. C.

[125] Ouyang Q., Zeng S., Jiang L., Hong L., Xu G., Dinh X.Q., Qian J., He S., Qu J., Coquet P. (2016). Sensitivity enhancement of transition metal dichalcogenides/silicon nanostructure-based surface plasmon resonance biosensor. Sci. Rep..

[126] Giovannetti G., Khomyakov P.A., Brocks G., Karpan V.v., van den Brink J., Kelly P.J. (2008). Doping graphene with metal contacts. Phys. Rev. Lett..

[127] Zeng S., Baillargeat D., Ho H.P., Yong K.T. (2014). Nanomaterials enhanced surface plasmon resonance for biological and chemical sensing applications. Chem. Soc. Rev..

[128] Chung K., Rani A., Lee J.E., Kim J.E., Kim Y., Yang H., Kim S.O., Kim D., Kim D.H. (2015). Systematic study on the sensitivity enhancement in graphene plasmonic sensors based on layer-by-layer self-assembled graphene oxide multilayers and their reduced analogues. ACS Appl. Mater. Interfaces.

[129] Xia S.X., Zhai X., Wang L.L., Wen S.C. (2018). Plasmonically induced transparency in double-layered graphene nanoribbons. Photonics Res..

[130] Xia S., Zhai X., Wang L., Xiang Y., Wen S. (2022). Plasmonically induced transparency in phase-coupled graphene nanoribbons. Phys. Rev. B.

[131] Xia S.X., Zhang D., Zhai X., Wang L.L., Wen S.C. (2023). Phase-controlled topological plasmons in 1D graphene nanoribbon array. Appl. Phys. Lett..

[132] Xia S.X., Zhang D., Zheng Z., Zhai X., Li H., Liu J.Q., Wang L.L., Wen S.C. (2023). Topological plasmons in stacked graphene nanoribbons. Opt. Lett..

[133] Xiao S., Wang T., Liu T., Zhou C., Jiang X., Zhang J. (2020). Active metamaterials and metadevices: A review. J. Phys. D Appl. Phys..

[134] Hu T., Mei X., Wang Y., Weng X., Liang R., Wei M. (2019). Two-dimensional nanomaterials: Fascinating materials in biomedical field. Sci. Bull..

[135] Wang P., Krasavin A.V., Liu L., Jiang Y., Li Z., Guo X., Tong L., Zayats A.V. (2022). Molecular plasmonics with metamaterials. Chem. Rev..

[136] Homola J., Koudela I., Yee S.S. (1999). Surface plasmon resonance sensors based on diffraction gratings and prism couplers: Sensitivity comparison. Sens. Actuators B Chem..

[137] Vukusic P., Bryan-Brown G., Sambles J. (1992). Surface plasmon resonance on gratings as a novel means for gas sensing. Sens. Actuators B Chem..

[138] Jory M., Vukusic P., Sambles J. (1994). Development of a prototype gas sensor using surface plasmon resonance on gratings. Sens. Actuators B Chem..

[139] Adam P., Dostálek J., Homola J. (2006). Multiple surface plasmon spectroscopy for study of biomolecular systems. Sensors Actuators B Chem..

[140] Monteiro J.P., Ferreira J., Sabat R.G., Rochon P., Santos M.J.L., Girotto E.M. (2012). SPR based biosensor using surface relief grating in transmission mode. Sens. Actuators B Chem..

[141] Dou X., Chung P.Y., Jiang P., Dai J. (2012). Surface plasmon resonance and surface-enhanced Raman scattering sensing enabled by digital versatile discs. Appl. Phys. Lett..

[142] Dou X., Phillips B.M., Chung P.Y., Jiang P. (2012). High surface plasmon resonance sensitivity enabled by optical disks. Opt. Lett..

[143] Chien F.C., Lin C.Y., Yih J.N., Lee K.L., Chang C.W., Wei P.K., Sun C.C., Chen S.J. (2007). Coupled waveguide–surface plasmon resonance biosensor with subwavelength grating. Biosens. Bioelectron..

[144] Lodewijks K., Ryken J., Van Roy W., Borghs G., Lagae L., Van Dorpe P. (2013). Tuning the Fano resonance between localized and propagating surface plasmon resonances for refractive index sensing applications. Plasmonics.

[145] Cetin A., Yanik A.A., Yilmaz C., Somu S., Busnaina A., Altug H. (2011). Monopole antenna arrays for optical trapping, spectroscopy, and sensing. Appl. Phys. Lett..

[146] Wang Y., Wu L., Wong T.I., Bauch M., Zhang Q., Zhang J., Liu X., Zhou X., Bai P., Dostalek J. (2016). Directional fluorescence emission co-enhanced by localized and propagating surface plasmons for biosensing. Nanoscale.

[147] Chen S., Meng L., Hu J., Yang Z. (2015). Fano interference between higher localized and propagating surface plasmon modes in nanovoid arrays. Plasmonics.

[148] Live L.S., Dhawan A., Gibson K.F., Poirier-Richard H.P., Graham D., Canva M., Vo-Dinh T., Masson J.F. (2012). Angle-dependent resonance of localized and propagating surface plasmons in microhole arrays for enhanced biosensing. Anal. Bioanal. Chem..

[149] Kelf T., Sugawara Y., Cole R., Baumberg J., Abdelsalam M., Cintra S., Mahajan S., Russell A., Bartlett P. (2006). Localized and delocalized plasmons in metallic nanovoids. Phys. Rev. B.

[150] Chang S.H., Gray S.K., Schatz G.C. (2005). Surface plasmon generation and light transmission by isolated nanoholes and arrays of nanoholes in thin metal films. Opt. Express.

[151] Tabatabaei M., Najiminaini M., Davieau K., Kaminska B., Singh M.R., Carson J.J., Lagugné-Labarthet F. (2015). Tunable 3D plasmonic cavity nanosensors for surface-enhanced Raman spectroscopy with sub-femtomolar limit of detection. Acs Photonics.

[152] Schwind M., Kasemo B., Zoric I. (2013). Localized and propagating plasmons in metal films with nanoholes. Nano Lett..

[153] Couture M., Live L.S., Dhawan A., Masson J.F. (2012). EOT or Kretschmann configuration? Comparative study of the plasmonic modes in gold nanohole arrays. Analyst.

[154] Hong X., Hall E.A. (2012). Contribution of gold nanoparticles to the signal amplification in surface plasmon resonance. Analyst.

[155] Špringer T., Homola J. (2012). Biofunctionalized gold nanoparticles for SPR-biosensor-based detection of CEA in blood plasma. Anal. Bioanal. Chem..

[156] Li R., Feng F., Chen Z.Z., Bai Y.F., Guo F.F., Wu F.Y., Zhou G. (2015). Sensitive detection of carcinoembryonic antigen using surface plasmon resonance biosensor with gold nanoparticles signal amplification. Talanta.

[157] Yang C.T., Wu L., Bai P., Thierry B. (2016). Investigation of plasmonic signal enhancement based on long range surface plasmon resonance with gold nanoparticle tags. J. Mater. Chem. C.

[158] Reinhard B.M., Sheikholeslami S., Mastroianni A., Alivisatos A.P., Liphardt J. (2007). Use of plasmon coupling to reveal the dynamics of DNA bending and cleavage by single EcoRV restriction enzymes. Proc. Natl. Acad. Sci. USA.

[159] Dreaden E.C., Alkilany A.M., Huang X., Murphy C.J., El-Sayed M.A. (2012). The golden age: Gold nanoparticles for biomedicine. Chem. Soc. Rev..

[160] Kretschmer F., Muehlig S., Hoeppener S., Winter A., Hager M.D., Rockstuhl C., Pertsch T., Schubert U.S. (2014). Survey of plasmonic nanoparticles: From synthesis to application. Part. Part. Syst. Charact..

[161] Nordlander P., Oubre C., Prodan E., Li K., Stockman M. (2004). Plasmon hybridization in nanoparticle dimers. Nano Lett..

[162] Jain P.K., El-Sayed M.A. (2010). Plasmonic coupling in noble metal nanostructures. Chem. Phys. Lett..

[163] Unser S., Bruzas I., He J., Sagle L. (2015). Localized surface plasmon resonance biosensing: Current challenges and approaches. Sensors.

[164] Aldewachi H., Chalati T., Woodroofe M., Bricklebank N., Sharrack B., Gardiner P. (2018). Gold nanoparticle-based colorimetric biosensors. Nanoscale.

[165] Zagorovsky K., Chan W.C. (2013). A plasmonic DNAzyme strategy for point-of-care genetic detection of infectious pathogens. Angew. Chem. Int. Ed..

[166] De La Rica R., Stevens M.M. (2013). Plasmonic ELISA for the detection of analytes at ultralow concentrations with the naked eye. Nat. Protoc..

[167] Rong G., Wang H., Reinhard B.M. (2010). Insights from a nanoparticle minuet: Two-dimensional membrane profiling through silver plasmon ruler tracking. Nano Lett..

[168] Lee S.E., Chen Q., Bhat R., Petkiewicz S., Smith J.M., Ferry V.E., Correia A.L., Alivisatos A.P., Bissell M.J. (2015). Reversible aptamer-Au plasmon rulers for secreted single molecules. Nano Lett..

[169] Chen J.I., Durkee H., Traxler B., Ginger D.S. (2011). Optical detection of protein in complex media with plasmonic nanoparticle dimers. Small.

[170] Smekal A. (1923). Zur quantentheorie der dispersion. Naturwissenschaften.

[171] Almehmadi L.M., Curley S.M., Tokranova N.A., Tenenbaum S.A., Lednev I.K. (2019). Surface enhanced Raman spectroscopy for single molecule protein detection. Sci. Rep..

[172] Wang H.N., Register J.K., Fales A.M., Gandra N., Cho E.H., Boico A., Palmer G.M., Klitzman B., Vo-Dinh T. (2018). Surface-enhanced Raman scattering nanosensors for in vivo detection of nucleic acid targets in a large animal model. Nano Res..

[173] Ding S.Y., Yi J., Li J.F., Ren B., Wu D.Y., Panneerselvam R., Tian Z.Q. (2016). Nanostructure-based plasmon-enhanced Raman spectroscopy for surface analysis of materials. Nat. Rev. Mater..

[174] Le Ru E.C., Blackie E., Meyer M., Etchegoin P.G. (2007). Surface enhanced Raman scattering enhancement factors: A comprehensive study. J. Phys. Chem. C.

[175] Solis D.M., Taboada J.M., Obelleiro F., Liz-Marzan L.M., Garcia de Abajo F.J. (2017). Optimization of nanoparticle-based SERS substrates through large-scale realistic simulations. ACS Photonics.

[176] Wang K., Sun D.W., Pu H., Wei Q. (2019). Shell thickness-dependent Au@ Ag nanoparticles aggregates for high-performance SERS applications. Talanta.

[177] Nie S., Emory S.R. (1997). Probing single molecules and single nanoparticles by surface-enhanced Raman scattering. Science.

[178] Michaels A.M., Jiang N., Brus L. (2000). Ag nanocrystal junctions as the site for surface-enhanced Raman scattering of single rhodamine 6G molecules. J. Phys. Chem. B.

[179] Atay T., Song J.H., Nurmikko A.V. (2004). Strongly interacting plasmon nanoparticle pairs: From dipole-dipole interaction to conductively coupled regime. Nano Lett..

[180] Gunnarsson L., Rindzevicius T., Prikulis J., Kasemo B., Käll M., Zou S., Schatz G.C. (2005). Confined plasmons in nanofabricated single silver particle pairs: Experimental observations of strong interparticle interactions. J. Phys. Chem. B.

[181] Marhaba S., Bachelier G., Bonnet C., Broyer M., Cottancin E., Grillet N., Lermé J., Vialle J.L., Pellarin M. (2009). Surface plasmon resonance of single gold nanodimers near the conductive contact limit. J. Phys. Chem. C.

[182] McMahon J.M., Li S., Ausman L.K., Schatz G.C. (2012). Modeling the effect of small gaps in surface-enhanced Raman spectroscopy. J. Phys. Chem. C.

[183] Wang P., Wu L., Lu Z., Li Q., Yin W., Ding F., Han H. (2017). Gecko-inspired nanotentacle surface-enhanced Raman spectroscopy substrate for sampling and reliable detection of pesticide residues in fruits and vegetables. Anal. Chem..

[184] Chauhan N., Saxena K., Jain U. (2022). Single molecule detection; from microscopy to sensors. Int. J. Biol. Macromol..

[185] Gauglitz G. (2020). ABC Spotlight on single-molecule detection. Anal. Bioanal. Chem..

[186] Melnychuk N., Egloff S., Runser A., Reisch A., Klymchenko A.S. (2020). Light-Harvesting Nanoparticle Probes for FRET-Based Detection of Oligonucleotides with Single-Molecule Sensitivity. Angew. Chem. Int. Ed..

[187] Akkilic N., Geschwindner S., Höök F. (2020). Single-molecule biosensors: Recent advances and applications. Biosens. Bioelectron..

[188] Guo H., Qian K., Cai A., Tang J., Liu J. (2019). Ordered gold nanoparticle arrays on the tip of silver wrinkled structures for single molecule detection. Sens. Actuators B Chem..

[189] Zong C., Premasiri R., Lin H., Huang Y., Zhang C., Yang C., Ren B., Ziegler L.D., Cheng J.X. (2019). Plasmon-enhanced stimulated Raman scattering microscopy with single-molecule detection sensitivity. Nat. Commun..

[190] Jaculbia R.B., Imada H., Miwa K., Iwasa T., Takenaka M., Yang B., Kazuma E., Hayazawa N., Taketsugu T., Kim Y. (2020). Single-molecule resonance Raman effect in a plasmonic nanocavity. Nat. Nanotechnol..

[191] Semeniak D., Cruz D.F., Chilkoti A., Mikkelsen M.H. (2023). Plasmonic Fluorescence Enhancement in Diagnostics for Clinical Tests at Point-of-Care: A Review of Recent Technologies. Adv. Mater..

[192] Kar A., Praneeth N., Khatua S., Datta B. (2023). Use of Single-Molecule Plasmon-Enhanced Fluorescence to Investigate Ligand Binding to G-Quadruplex DNA. J. Phys. Chem. Lett..

[193] Li C.Y., Duan S., Yi J., Wang C., Radjenovic P.M., Tian Z.Q., Li J.F. (2020). Real-time detection of single-molecule reaction by plasmon-enhanced spectroscopy. Sci. Adv..

[194] Spitzberg J.D., Zrehen A., van Kooten X.F., Meller A. (2019). Plasmonic-nanopore biosensors for superior single-molecule detection. Adv. Mater..

[195] Li W., Zhou J., Maccaferri N., Krahne R., Wang K., Garoli D. (2022). Enhanced optical spectroscopy for multiplexed DNA and protein-sequencing with plasmonic nanopores: Challenges and prospects. Anal. Chem..

[196] Shi X., Verschueren D.V., Dekker C. (2018). Active delivery of single DNA molecules into a plasmonic nanopore for label-free optical sensing. Nano Lett..

[197] Liu H.L., Ahmed S.A., Jiang Q.C., Shen Q., Zhan K., Wang K. (2023). Gold Nanotriangle-Assembled Nanoporous Structures for Electric Field-Assisted Surface-Enhanced Raman Scattering Detection of Adenosine Triphosphate. ACS Sens..

[198] Verschueren D.V., Pud S., Shi X., De Angelis L., Kuipers L., Dekker C. (2019). Label-free optical detection of DNA translocations through plasmonic nanopores. ACS Nano.

[199] Zhou J., Lan Q., Li W., Ji L.N., Wang K., Xia X.H. (2023). Single Molecule Protein Segments Sequencing by a Plasmonic Nanopore. Nano Lett..

[200] Chen C., Li Y., Kerman S., Neutens P., Willems K., Cornelissen S., Lagae L., Stakenborg T., Van Dorpe P. (2018). High spatial resolution nanoslit SERS for single-molecule nucleobase sensing. Nat. Commun..

[201] Huang J.A., Mousavi M.Z., Giovannini G., Zhao Y., Hubarevich A., Soler M.A., Rocchia W., Garoli D., De Angelis F. (2020). Multiplexed discrimination of single amino acid residues in polypeptides in a single SERS hot spot. Angew. Chem. Int. Ed..

[202] Li W., Zhou J., Lan Q., Ding X.L., Pan X.T., Ahmed S.A., Ji L.N., Wang K., Xia X.H. (2023). Single-Molecule Electrical and Spectroscopic Profiling Protein Allostery Using a Gold Plasmonic Nanopore. Nano Lett..

[203] Shi W., Friedman A.K., Baker L.A. (2017). Nanopore sensing. Anal. Chem..

[204] Assad O.N., Gilboa T., Spitzberg J., Juhasz M., Weinhold E., Meller A. (2017). Light-enhancing plasmonic-nanopore biosensor for superior single-molecule detection. Adv. Mater.

[205] Zambrana-Puyalto X., Ponzellini P., Maccaferri N., Garoli D. (2020). Förster-resonance energy transfer between diffusing molecules and a functionalized plasmonic nanopore. Phys. Rev. Appl..

[206] Klughammer N., Dekker C. (2021). Palladium zero-mode waveguides for optical single-molecule detection with nanopores. Nanotechnology.

[207] Barulin A., Claude J.B., Patra S., Bonod N., Wenger J. (2019). Deep ultraviolet plasmonic enhancement of single protein autofluorescence in zero-mode waveguides. Nano Lett..

[208] Pang Y., Gordon R. (2012). Optical trapping of a single protein. Nano Lett..

[209] Belkin M., Chao S.H., Jonsson M.P., Dekker C., Aksimentiev A. (2015). Plasmonic nanopores for trapping, controlling displacement, and sequencing of DNA. ACS Nano.

[210] Rogez B., Marmri Z., Thibaudau F., Baffou G. (2021). Thermoplasmonics of metal layers and nanoholes. APL Photonics.

[211] Lu J., Yang H., Zhou L., Yang Y., Luo S., Li Q., Qiu M. (2017). Light-induced pulling and pushing by the synergic effect of optical force and photophoretic force. Phys. Rev. Lett..

[212] Lin L., Wang M., Peng X., Lissek E.N., Mao Z., Scarabelli L., Adkins E., Coskun S., Unalan H.E., Korgel B.A. (2018). Opto-thermoelectric nanotweezers. Nat. Photonics.

[213] Hong C., Yang S., Ndukaife J.C. (2020). Stand-off trapping and manipulation of sub-10 nm objects and biomolecules using opto-thermo-electrohydrodynamic tweezers. Nat. Nanotechnol..

[214] Zhao Y., Iarossi M., De Fazio A.F., Huang J.A., De Angelis F. (2022). Label-free optical analysis of biomolecules in solid-state nanopores: Toward single-molecule protein sequencing. ACS Photonics.

[215] Shalabney A., Abdulhalim I. (2011). Sensitivity-enhancement methods for surface plasmon sensors. Laser Photonics Rev..

[216] Shalabney A., Abdulhalim I. (2010). Electromagnetic fields distribution in multilayer thin film structures and the origin of sensitivity enhancement in surface plasmon resonance sensors. Sens. Actuators A Phys..

[217] Chen Z., Liu L., He Y., Ma H. (2016). Resolution enhancement of surface plasmon resonance sensors with spectral interrogation: Resonant wavelength considerations. Appl. Opt..

[218] Akib T.B.A., Mou S.F., Rahman M.M., Rana M.M., Islam M.R., Mehedi I.M., Mahmud M.P., Kouzani A.Z. (2021). Design and numerical analysis of a graphene-coated SPR biosensor for rapid detection of the novel coronavirus. Sensors.

[219] Sharma P.K., Kumar J.S., Singh V.V., Biswas U., Sarkar S.S., Alam S.I., Dash P.K., Boopathi M., Ganesan K., Jain R. (2020). Surface plasmon resonance sensing of Ebola virus: A biological threat. Anal. Bioanal. Chem..

[220] Moznuzzaman M., Khan I., Islam M.R. (2021). Nano-layered surface plasmon resonance-based highly sensitive biosensor for virus detection: A theoretical approach to detect SARS-CoV-2. AIP Adv..

[221] Dai Z., Xu X., Wang Y., Li M., Zhou K., Zhang L., Tan Y. (2022). Surface plasmon resonance biosensor with laser heterodyne feedback for highly-sensitive and rapid detection of COVID-19 spike antigen. Biosens. Bioelectron..

[222] Omar N.A.S., Fen Y.W., Abdullah J., Mustapha Kamil Y., Daniyal W.M.E.M.M., Sadrolhosseini A.R., Mahdi M.A. (2020). Sensitive detection of dengue virus type 2 E-proteins signals using self-assembled monolayers/reduced graphene oxide-PAMAM dendrimer thin film-SPR optical sensor. Sci. Rep..

[223] Xu H., Song Y., Zhu P., Zhao W., Liu T., Wang Q., Zhao T. (2021). Alcohol sensor based on surface plasmon resonance of ZnO nanoflowers/Au structure. Materials.

[224] Rahman M.M., Rana M.M., Rahman M.S., Anower M., Mollah M.A., Paul A.K. (2020). Sensitivity enhancement of SPR biosensors employing heterostructure of PtSe2 and 2D materials. Opt. Mater..

